# Insights on correlation dimension from dynamics mapping of three experimental nonlinear laser systems

**DOI:** 10.1371/journal.pone.0181559

**Published:** 2017-08-24

**Authors:** Christopher J. McMahon, Joshua P. Toomey, Deb M. Kane

**Affiliations:** MQ Photonics Research Centre and Department of Physics & Astronomy, Macquarie University, Sydney, NSW, Australia; Technical University of Madrid, SPAIN

## Abstract

**Background:**

We have analysed large data sets consisting of tens of thousands of time series from three Type B laser systems: a semiconductor laser in a photonic integrated chip, a semiconductor laser subject to optical feedback from a long free-space-external-cavity, and a solid-state laser subject to optical injection from a master laser. The lasers can deliver either constant, periodic, pulsed, or chaotic outputs when parameters such as the injection current and the level of external perturbation are varied. The systems represent examples of experimental nonlinear systems more generally and cover a broad range of complexity including systematically varying complexity in some regions.

**Methods:**

In this work we have introduced a new procedure for semi-automatically interrogating experimental laser system output power time series to calculate the correlation dimension (CD) using the commonly adopted Grassberger-Proccacia algorithm. The new CD procedure is called the ‘minimum gradient detection algorithm’. A value of minimum gradient is returned for all time series in a data set. In some cases this can be identified as a CD, with uncertainty.

**Findings:**

Applying the new ‘minimum gradient detection algorithm’ CD procedure, we obtained robust measurements of the correlation dimension for many of the time series measured from each laser system. By mapping the results across an extended parameter space for operation of each laser system, we were able to confidently identify regions of low CD (CD < 3) and assign these robust values for the correlation dimension. However, in all three laser systems, we were not able to measure the correlation dimension at all parts of the parameter space. Nevertheless, by mapping the staged progress of the algorithm, we were able to broadly classify the dynamical output of the lasers at all parts of their respective parameter spaces. For two of the laser systems this included displaying regions of high-complexity chaos and dynamic noise. These high-complexity regions are differentiated from regions where the time series are dominated by technical noise. This is the first time such differentiation has been achieved using a CD analysis approach.

**Conclusions:**

More can be known of the CD for a system when it is interrogated in a mapping context, than from calculations using isolated time series. This has been shown for three laser systems and the approach is expected to be useful in other areas of nonlinear science where large data sets are available and need to be semi-automatically analysed to provide real dimensional information about the complex dynamics. The CD/minimum gradient algorithm measure provides additional information that complements other measures of complexity and relative complexity, such as the permutation entropy; and conventional physical measurements.

## 1. Introduction

### 1.1 Correlation dimension

In the science of chaos and nonlinear dynamics, fractal dimensions are used as a quantitative measure of a system’s complexity [1, 2]. The correlation dimension (CD) is one such fractal dimension and it is the one most widely used in analysis of experimental nonlinear systems [3]. It is calculated after applying the Grassberger-Proccacia algorithm to a time series tracing the behaviour of the system over a finite time interval [4]. The benefit of the CD calculation as an analytical tool for nonlinear science is that it classifies different dynamical behaviours with a single number. The larger the number, the more complex the system dynamics. That is, more variables are required to model the system [3, 5]. A simple periodic time series will have a CD equal to 1. Such a time series with any number of harmonic overtones should also have a CD of 1. A time series with two periodic components of incommensurate frequencies should have a CD equal to 2 [2]. The classical example of a system displaying chaotic dynamics, the Lorenz attractor, has a fractional CD of approximately 2.05. The non-integer dimension indicates that the Lorenz system evolves chaotically, with a phase space attractor that is a hybrid between a surface and a 3-D spatial structure [1]. Noise, which has no underlying dynamical structure, effectively has an infinite CD.

CD measurements have been applied in a broad range of scientific fields to interrogate systems displaying chaotic dynamics. The CD has been used to quantify the complexity of sounds from musical instruments [1, 2], to study regular and irregular patterns in the magnitude of light emitted from stars and other astronomical objects [6] and to track movements in indicators within financial markets [7]. Chaotic behaviour can also be induced in electronic circuits. The Grassberger-Proccacia algorithm has been applied, for example, to characterise regular, chaotic and random behaviour in an electronic model of a ball bouncing on an oscillating table [8]. It has applications in mechanical systems, for instance in the analysis of vibrations from gear boxes, where changes in the CD identified the formation of fatigue cracks within the gears [9]. It has been applied in a large number of investigations within various medical science fields, where electrophysical signals are measured. For example, irregular patterns of heart behaviour have been detected within electrocardiograms [10, 11]. It was found to be a useful tool in studying electroencephalograms from patients exposed to different sets of visual stimuli [3]. The CD was also found to be useful in quantifying differences between the electroencephalograms and magnetoencephalograms from patients with healthy brain states and those with Parkinson’s disease, Alzheimer’s disease [12, 13] and schizophrenia [14].

Although alternatives to the Grassberger-Proccacia (G-P) algorithm exist, it has been, and still is, extensively utilised. The limitations of the G-P algorithm are also well recognised, particularly its sensitivity to time series with moderate to large noise components [6]. However, because it is a well understood method and its use is so ubiquitous we have chosen to continue to use it and to research how successful it can be in studies of the dynamics of experimental nonlinear systems, laser systems in our case. This research gives significant insights into how to interpret CD values derived using the G-P algorithm on such data. These insights come in part from CD mapping the output power dynamics from the three different nonlinear laser systems, and contrasts in and between the systems which have informed a more robust interpretation of all the results. The theory and process of calculating CD is described in detail in Section A in S1 File, “Calculating the correlation dimension of a time series using the Grassberger-Proccacia algorithm”. After a time series is processed by the G-P algorithm values for the ‘correlation sum’ as a function of ‘hypersphere radii’ are generated [1, 2, 4]. The calculation is repeated for incrementing ‘embedding dimensions’. The rate of change of the correlation sum as a function of hypersphere radius is the quantity of interest in this analysis. When this gradient saturates for increasing embedding dimensions, over a range of radii, it is referred to as a scaling region. If a scaling region can be clearly identified from the analysis of a single time series [10, 11, 15] the gradient in this region can be robustly assigned as the CD. In the analysis of noise-free simulated data, such scaling regions may be identified with ease. But in the case of experimental data, noise is usually present in some form and can conceal or almost conceal a scaling region which would otherwise exist [6].

### 1.2 Nonlinear laser systems

Laser systems are excellent for studies in nonlinear science, because the experimenter has a high degree of control over the time series length, sampling rate and signal-to-noise ratio [15]. They also have many practical applications due to their ability to deliver chaotic outputs [16]. Lasers fall into three classes: A, B and C [17]. Class C lasers are described by three differential equations for electric field, polarisation and population inversion. All three variables have similar relaxation times in a Class C laser. They are mainly based on gaseous lasing mediums, such as ammonia, and output light in the far-infrared [18]. The oscillations of Class A lasers, including dye and He-Ne lasers, can be described by a single differential equation because the relaxation rates for polarisation and population inversion are much faster than for the electric field and, therefore, their equations can be adiabatically eliminated. They deliver the most stable outputs. Class B lasers, including solid-state lasers, semiconductor lasers and CO_2_ lasers, can be described by two rate equations [17] as only the fast relaxing polarisation can be eliminated. Thus, they are intrinsically stable [19]. However, with the increase in the number of degrees of freedom in the system when subject to an external perturbation, the output can become chaotic. Such perturbations can include direct modulation of laser operating parameters such as the injection current [20], optical injection of light from another laser [21] or optical feedback, where light is reflected back into a laser from an external mirror [17, 22–24]. Class B lasers have many practical uses because a number of system parameters can be tuned to deliver the type of output that an operator desires: continuous wave (CW), oscillatory, pulsed, or chaotic outputs [25, 26]. This paper examines the application of CD analysis to time series from three Class B lasers subjected to perturbations: an optically injected solid-state laser (OISSL), a semiconductor laser with optical feedback (SLwOF) from an external mirror, and a photonic integrated chip (PIC) semiconductor laser with optical feedback.

We have previously reported on the output dynamics generated by these three laser systems, by mapping characteristics of the time series measured across wide operating parameter ranges [15, 27–34]. For example, for the semiconductor laser with optical feedback from an external mirror (SLwOF), fine adjustments were made to the current injected into the laser diode and/or the fraction of light reflected into the laser from the external mirror. Tens of thousands of time series were measured and maps were produced of time series characteristics such as the RMS amplitude and significant features of the autocorrelation function [29, 30]. Maps of such a simple measurement can provide guidance on areas of the parameter space where the dynamics change, but they do not allow for direct discrimination between dynamics. A chaotic time series and a pulsing time series may have the same RMS amplitude. A computationally efficient measurement of relative complexity can be obtained from the permutation entropy of a time series [35]. The permutation entropy quantifies the distribution of ordinal patterns present in a time series. It scales between 0 and 1, with low numbers indicating that there are many repeated patterns in a time series, which implies low complexity. High permutation entropy values imply uniform distribution of all possible patterns and therefore highly complex, unpredictable dynamics. When the permutation entropy of many time series is mapped, this provides an informative relative measurement of complexity [29, 30]. However, it is not possible to say that periodic time series, for example, should have a specific value for the permutation entropy. This is why the additional computational effort of calculating the CD may be worthwhile, because it offers the possibility of unequivocally categorising the dynamics of time series across the full parameter space of a laser system. The aim of the work presented herein was to interrogate the success of the CD measurement algorithm in categorising the dynamics of the three laser systems.

## 2. Time series measurement

The output power time series data from the three laser systems described below are all available from a publicly available open science web data base [36]. A summary of the time series datasets that are used to generate the dynamical maps here-in are presented in Table 1. It should also be noted that the specific details of how the experiment is sequenced and carried out is relevant to avoiding or minimising the effects of multistability. The maps may change in some of the detail if a different experimental sequence is followed.

**Table 1:** Summary of the datasets for each of the laser systems [36].

	Laser System & the Two Parameters on the Axes of the Dynamic Maps		
	Photonic Integrated Chip Laser (PIC) 1. DFB Injection current (15–50 mA) 2. Optical feedback (with gain: 0-10mA, with attenuation: 0- -2V)	Semiconductor laser with optical feedback (SLwOF) 1. Injection Current (45–74 mA) 2. Optical feedback (6.6% - 75%)	Optically injected solid state laser (OISSL) 1. Frequency detuning (-14.4–11.4 MHz) 2. Injection strength (0–4.5)*
Number of Values of each parameter in the dataset	1. 251; 2. Gain—101, Attenuation—21	1. 251; 2. 351	1. 315; 2. 176**
Number of time series	Gain– 35,451; Attenuation—7371	88,101	55,400
Number of data points in each time series (analysed)	80,000 (10,000)^+^	20,000 (10,000) ^+^	5000 (5000)
Sampling period	25 ps	50 ps	10 ns

*as normalised to the injection strength which causes injection locking at a frequency detuning.

**injection strength is ramped but is essentially constant in each of the 176 sections

^**+**^using more data points does not change the results

Issues of the signal to noise of the output power time series are common to all three systems. The term fundamental and technical noise is used to refer to the variations over time of the detected signal which is present when detecting the laser below threshold or the laser operating at a constant power. This includes spontaneous emission noise and all the fundamental and technical noise sources associated with the laser, the photodetection, and the oscilloscope. This noise is present on all detected output power time series but is generally of very small amplitude compared to the signal level associated with the nonlinear dynamics observed in the systems. Complex chaos and dynamical noise is used to describe the laser system operating in a region where deterministic chaos is expected as informed, at least qualitatively, from the rate equation or time delay differential equation model relevant to the particular system. Both the terms, chaos and dynamical noise, are used to cover the possibility that the output power, when operating in a chaotic region may have a dynamical component of noise as a consequence of the chaotic operation.

### 2.1 Photonic integrated chip (PIC) laser

**Fig 1:**
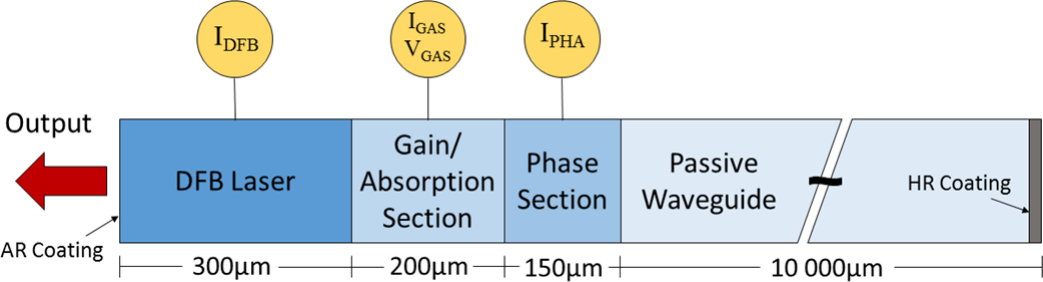
Schematic diagram of a four-section photonic integrated chip (PIC) laser. The InGaAsP DFB laser is 300 μm long. The gain/absorption section and the phase control section are 200 μm and 150 μm long, respectively. The passive waveguide is 10 mm long [25, 37]. Reprinted from [32] Opt Express vol. 23, no. 14, pp. 18754–62, 2015 under a CC BY license, with permission from OSA, original copyright 2015.

The construction of the four section PIC laser is shown schematically in Fig 1 and the device and packaging are described fully in [25, 37]. The system consists of a 300 μm long InGaAsP DFB laser with an operating wavelength of 1561 nm, a 200 μm gain or absorption (G/A) section (forward biased for gain, reverse biased for absorption), a 150 μm phase control section, and a 10 mm passive waveguide. The external facet of the passive waveguide is high reflectance (HR) coated. The individual device sections were SMA connectorised via micro-strip lines. The chip was connected to an output optical fiber via a tapered end at the anti-reflection (AR) coated end facet of the DFB section. Aposotolos Argyris of the National and Kapodistrian University of Athens used an automated data collection system to obtain time series measurements [25, 32]. In the maps presented herein, the phase section bias was set to 0 mA and the DFB injection current was stepped through a range of 35 mA, beginning at 15.0 mA, in 0.1 mA steps. The gain section current was stepped through a 10 mA range in 0.1 mA steps, beginning at 0 mA. This amplified the feedback light from the passive waveguide section. The optical feedback was also attenuated by applying a reverse bias voltage of 0 to 2 V in 0.1 V steps, across the G/A section so that it acted as an absorber section. For reverse bias voltages above 1 V there is essentially no feedback from the passive waveguide section. We note here that there is a range of feedback values that cannot be accessed when switching from forward bias to reverse bias of the G/A section. For each pair of operating parameters, the laser output power was sampled at 40 GSa/s for 2 μs (sampling interval 25 ps). The 12 GHz bandwidth of the real-time oscilloscope was the limiting bandwidth of the detection system. Each time series is 80 000 points long and there are 35 451 data points in the optical-feedback-with-gain region of the map, and 7371 in the optical-feedback-with-attenuation region.

**Fig 2:**
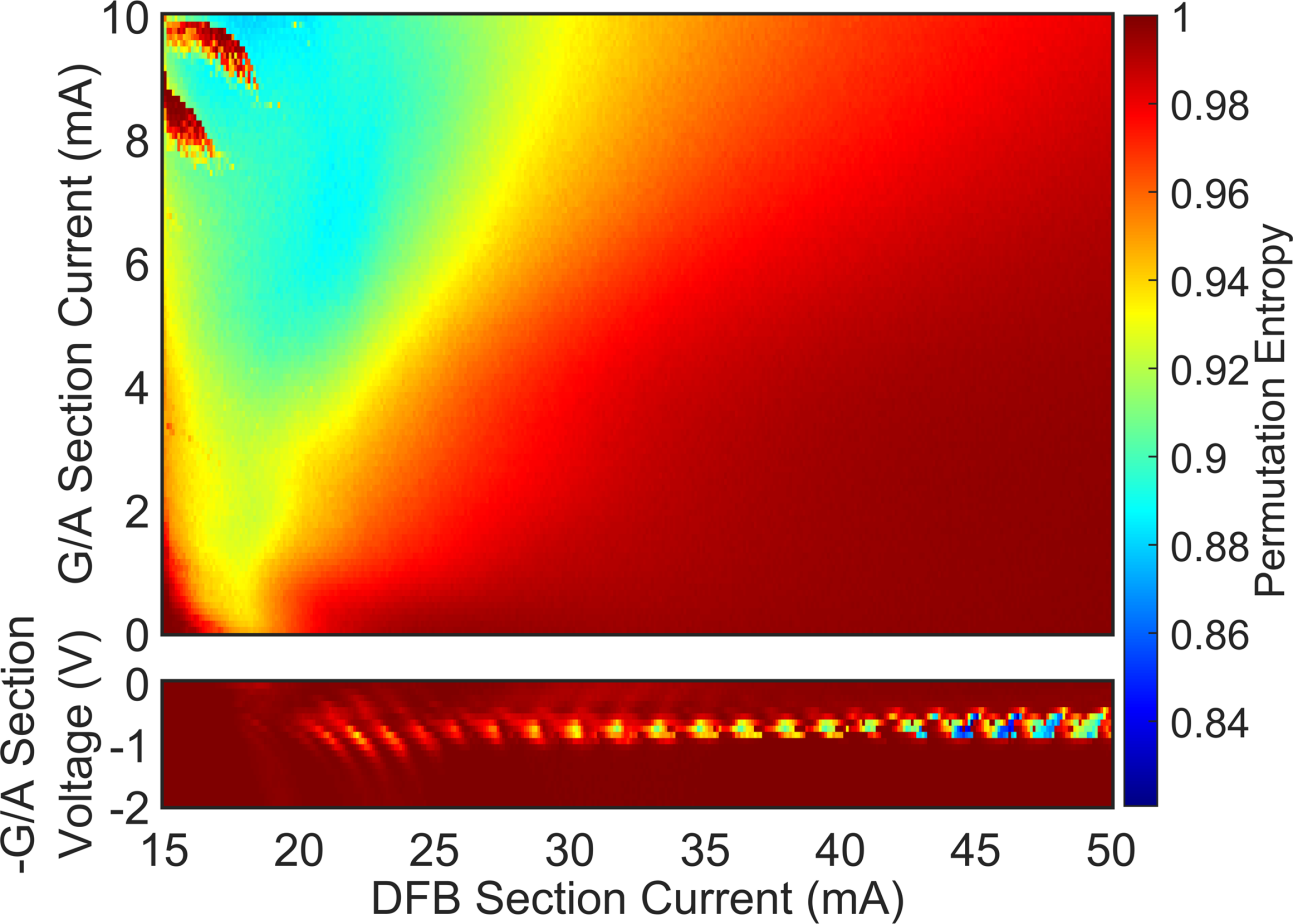
Permutation entropy map for the PIC laser. Permutation entropy as a function of DFB laser injection current and gain/absorption section current/bias, for a fixed phase section current = 0 mA. Calculated using an order of 3 and a time delay of 0.25 ns (10 points). Reprinted from [34] J. Lightwave Technology vol. 35, no.1, 88–95, 2017 under a CC BY license, with permission from IEEE, original copyright 2017.

Mapping of the dynamics of the PIC within the specified parameter space has been completed using permutation entropy (PE) as a relative measure of complexity [34]. Fig 2 shows the PE map where high values of PE correspond to higher levels of complexity. However, the PE measure approaches 1 for an infinitely long time series of white noise so this measure is not a sensitive one for differentiating higher dimensional nonlinear dynamics. Nor does it give quantitative information about the nature of the variation with time. Fig 2 does demonstrate that the complexity of the output power time series varies systematically with DFB injection current and gain section current, in what would normally be called a coherence collapse or chaotic region, for this system [17, 22]. Also, the dataset has been used to investigate the transition from the short cavity to the long cavity regime of a semiconductor laser with optical feedback system by contrasting regular pulse package output with broadband chaotic output [31]. These results suggest the dataset has regions of complexity of CD below 3 which have a reasonable prospect of being amenable to CD analysis.

### 2.2 Semiconductor laser with delayed optical feedback (SLwOF)

**Fig 3:**
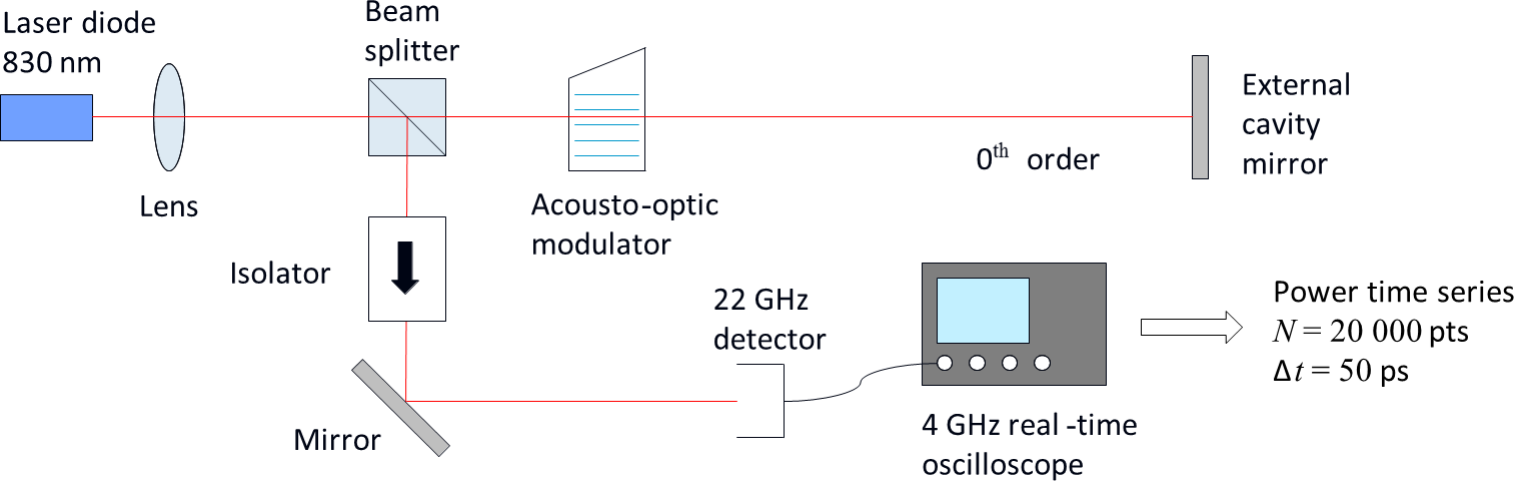
Semiconductor laser with delayed optical feedback system and layout of the measurement equipment. The optical feedback fraction is varied by increasing the rf drive to the acousto-optic modulator (AOM) which transfers more of the beam power to the first order diffracted beam which is blocked [30]. The output power is detected with a 22 GHz photodiode and time series are recorded using a digital oscilloscope with 4 GHz real-time bandwidth. Reprinted from Opt. Express vol. 22, no. 2, pp. 1713–25, 2014 under a CC BY license, with permission from OSA, original copyright 2014.

The experimental setup of a semiconductor laser with delayed optical feedback (SLwOF) from a long free-space external cavity is shown in Fig 3 [30]. It consisted of a multiple quantum well 830 nm semiconductor laser (APL 830–40) and a high reflectance external cavity mirror that provided the optical feedback. The laser output beam was collimated with an 8 mm focal length aspheric lens and then passed through a 50:50 cube beamsplitter. An acousto-optic modulator (G&H 23080) was used to control the fraction of the zeroth order beam that was reflected back into the laser. The beam splitter directs half the laser output into a 22 GHz photodiode. The photodiode signal was sampled at 20 GSa/s for 1 μs (sampling interval 50 ps) by a digital oscilloscope (Agilent Infiniium 54854A DSO), with a 4 GHz real-time bandwidth.

Output power time series were collected while stepping through values of the laser injection current and the fraction of delayed optical feedback. The injection current was swept from 45 mA to 70 mA in 0.1 mA steps. The zeroth order transmission of the AOM, which is proportional to the optical feedback level, was adjusted in 351 non-uniform steps between 75.5% and 6.5%. Each of the 88 101 time series that were measured consist of 20 000 points.

**Fig 4:**
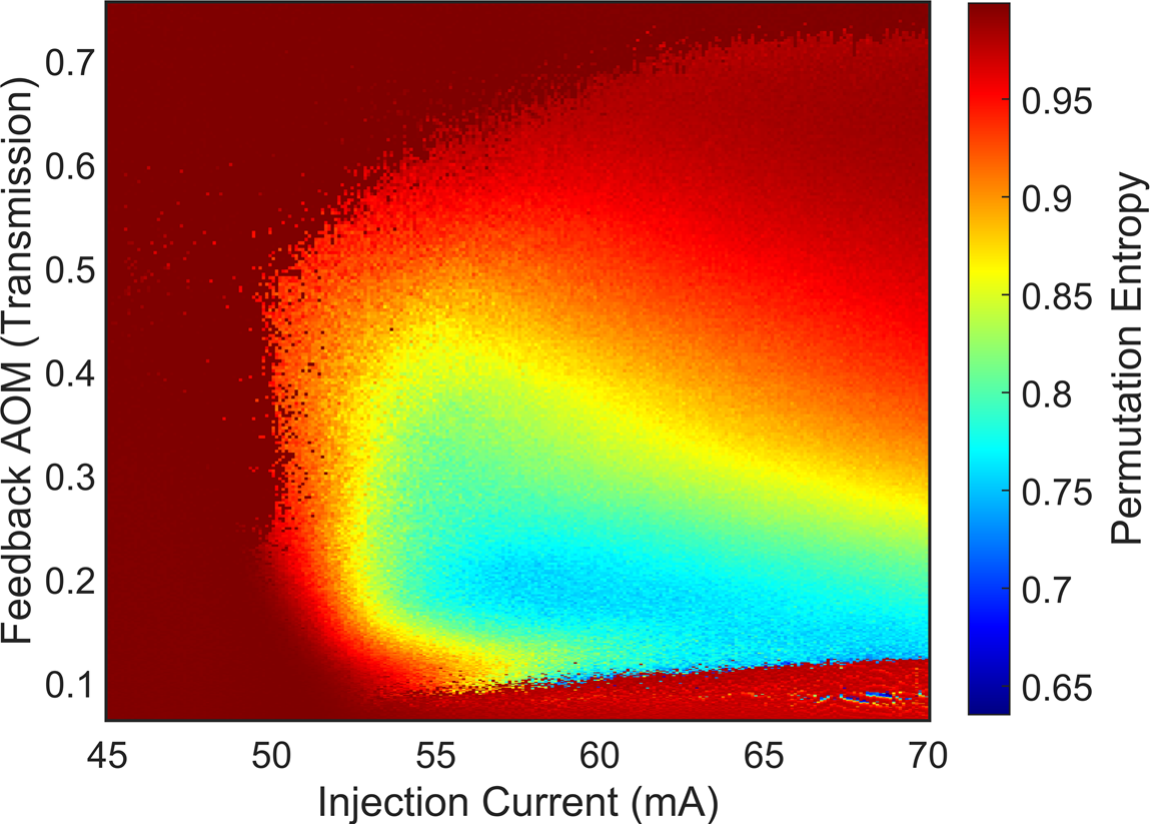
Permutation entropy map for semiconductor laser with delayed optical feedback system. The permutation entropy was interrogated with order 5 at a delay of 2 points or 0.1 ns. Reprinted from Opt Express vol. 22, no. 2, pp. 1713–25, 2014 under a CC BY license, with permission from OSA, original copyright 2014.

The dynamics of the SLwOF system have been analysed using permutation entropy. The results are reproduced in Fig 4 [30]. The map shows very high PE associated with the noise on the AC-coupled, photodetected-constant-output-power surrounding the lower PE coherence collapse region. The low feedback boundary of this region is where low frequency fluctuation (LFF) behaviour is observed [17, 22]. Again, it is seen that the complexity varies systematically in the coherence collapse region. The chaotic output power time series from this system are expected to be of high dimension and as such are not good candidates for CD analysis. Previous research [31] did not lead to robust CD values being found, although islands of periodic behaviour in the parameter space of a semiconductor laser with both optical feedback and a component of sinusoidal modulation of the injection current were successfully identified using CD analysis [31]. We show in the CD analysis reported here-in that increased understanding of the process of calculating the CD allows more to be learned on the quantified complexity of this system than has been possible before.

### 2.3 Optically injected solid state laser (OISSL)

**Fig 5:**
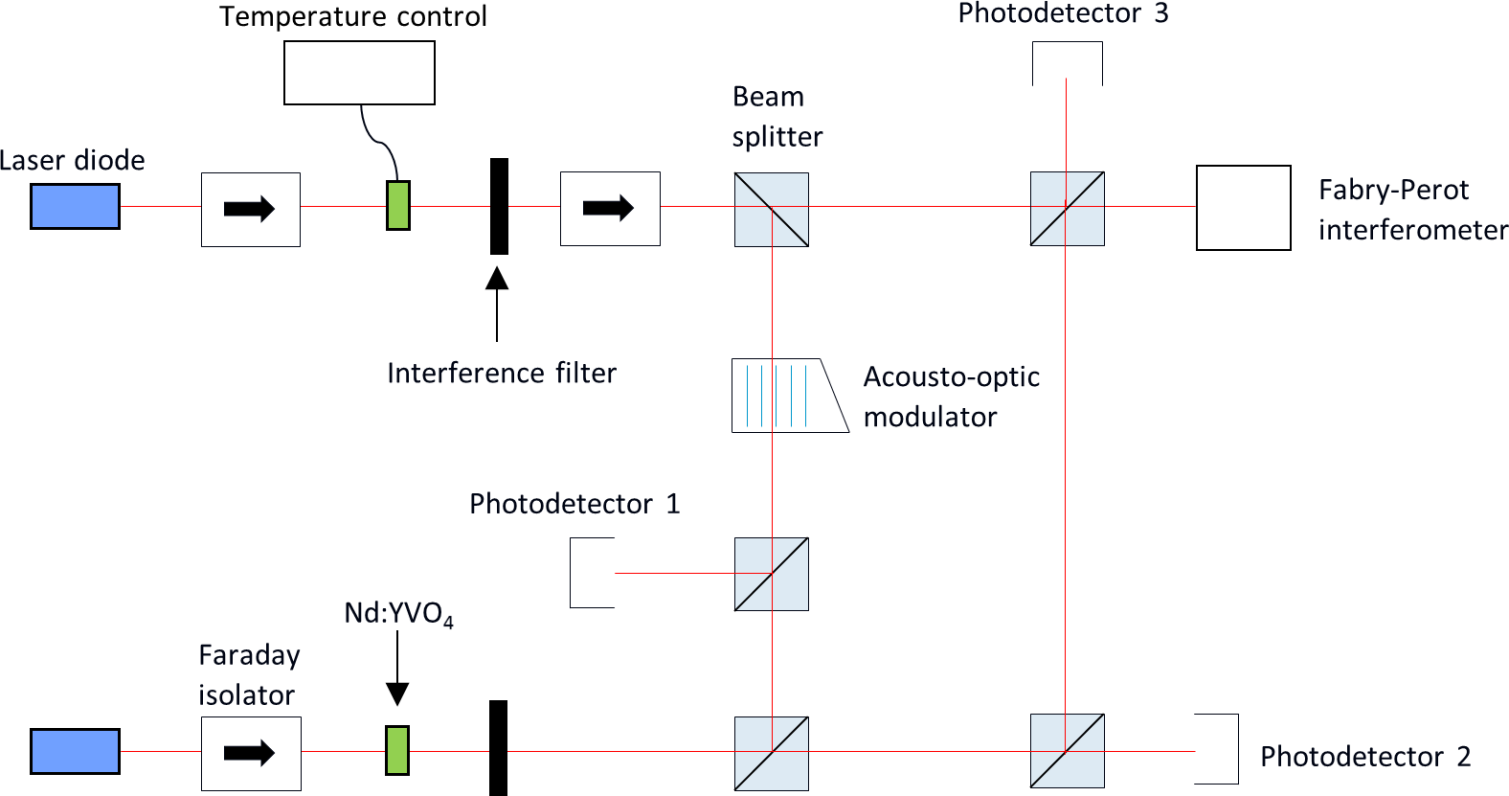
Setup of master and optically injected slave Nd:YVO_4_ solid state lasers. Light was injected from the master laser through an AOM into the slave laser to control the injected power. The temperature of the master laser was adjusted to set the frequency detuning between the master and slave lasers [15, 38].

This nonlinear laser system has multiple attractors and produces a dynamic map with several distinct types of dynamics. The particular implementation in Fig 5 consists of two Nd:YVO_4_ lasers (emission wavelength 1064 nm) operated in a master-slave configuration. The laser crystal cavities were formed by a HR coating (at 1064 nm) on the surface at the pumped facet (AR coated at the pumping wavelength) and a 5% output coupling at the end facet of the 1.0 mm long crystals (supplied by CASIX, with 1% Nd^3+^ atomic doping). The master laser was temperature controlled with a Peltier element. Two 150 W, 809 nm laser diodes were used to pump the solid state lasers. Feedback to the pump lasers was blocked using optical (Faraday rotation-based) isolators. Pump light which was not absorbed in the crystals was blocked from further propagation using interference filters. An additional Faraday isolator was placed in the beam path between the master and slave lasers to prevent optical feedback into the master laser. Light was injected from the master into the slave laser through an AOM to control the injected power. The relative injection power was measured on the photodetector PD1. The beat frequency between the lasers was measured on a 400 MHz photodetector PD3. The intensity time series of the slave laser was measured at PD2, which was connected to a Tektronix CSA 7404 oscilloscope.

The power of the injected beam was controlled via the rf drive level to the AOM (the first order, frequency shifted beam was used). The injection power was measured in arbitrary units by monitoring the amount of light before it was injected into the laser. The coupling constant of the injected field was unknown, so the actual injection strength was not known. The injection strength was represented here by the quantity *K*, which normalised the experimentally measured locking boundary against the boundary from a numerical model [39, 40]. The temperature of the master laser was adjusted to control the frequency detuning (*Δω*) between the lasers, which was measured from the beat frequency. The laser output intensity was recorded over time for a fixed level of detuning. The injection strength (*K*) was slowly increased from zero until frequency locking occurred between the master and slave lasers. Locking was eventually achieved for any detuning, as long as the injection power was high enough. The injection strength at which this occurs increases linearly with *Δω*. The injection sweep was repeated for 315 detuning values. In the maps presented herein the values for *Δω* have been scaled to lie in the range -4.11 to 3.25 times the 3.5 MHz angular relaxation oscillation frequency of the injected laser [38]. Each of the laser power time series contained 883 000 points, as measured, with a sampling interval of 10 ns. The time series were later split into 176 subsets of 5000 points. This is equivalent to a duration of 50 μs, which is much shorter than the 9 ms sweep of the injection strength. Thus, these time series subsets are a record of the dynamics of the slave laser at an essentially constant injection strength [15].

**Fig 6:**
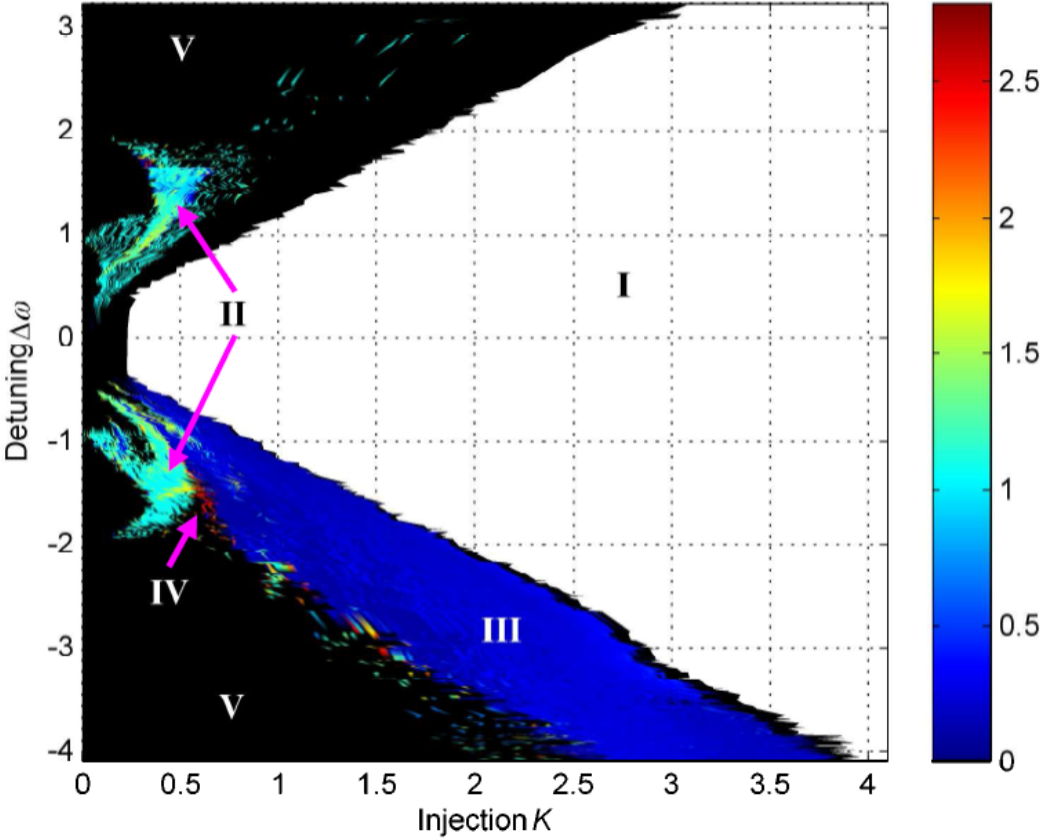
Correlation dimension map for the experimental master and optically injected slave Nd:YVO_4_ solid state lasers. Correlation dimension is mapped for an optical injection and frequency detuning parameter space (*K*, *Δω*). Different dynamical regions identified as I: injection locked; II: periodic; III: ‘spiky’ output; IV: chaotic; V: CW represented as black in this map. Reprinted from [15] Opt Express vol. 17, no. 9, pp. 7592–7608, 2009 under a CC BY license, with permission from OSA, original copyright 2009.

A previous study of measuring the CD for this dataset has been published which used the first generation algorithm for automatic CD calculation [15]. The experiment was carried out in a manner to control the system to remain on the same attractors throughout. The map shows regions of period doubling, and chaotic behaviour. Hopf, torus and saddle node bifurcations are shown from the theoretical bifurcation model of the system [40] and these bifurcations align with the boundaries between different dynamical regions in the map shown in Fig 6. This dataset has been shown in [15] to allow successful determination of CD values for some regions of the map. The values of CD between 0 and 1 found in region III are in need of understanding and this will be the subject of another paper. The minimum gradient detection algorithm developed and used in this work leads to a meaningful improvement in the CD mapping for this system.

## 3. Process for mapping the correlation dimension (CD) of laser output power time series

The theory behind the procedure for performing calculations using the Grassberger-Proccacia algorithm is well known and has been reported elsewhere [1, 2, 4, 5, 10, 11, 15, 41]. An overview is provided in Section A in S1 File. In summary, the first step of the process is to plot the phase space attractor for the nonlinear system. The true attractor can be displayed when time series for each system variable are plotted against each other; the number of axes must be equal to the number of system variables. In practice it is common that only one variable can be measured, such as the light output power from a laser, meaning that the true attractor cannot be plotted. Instead, a ‘reconstructed attractor’, with the same properties as the true attractor, is formed by plotting one time series against a time-delayed version of itself in two more dimensions [41]. The number of time series plotted, the original and several incrementally time-delayed versions, is called the ‘embedding dimension’. In this work, calculations were performed for embedding dimensions, *m*, between 5 and 10. We chose to use the position of the first local minimum of the mutual information function to set the appropriate delay time for each time series [15, 42]. Correlation sums were calculated using the TISEAN 3.0.1 programme [43].

**Fig 7:**
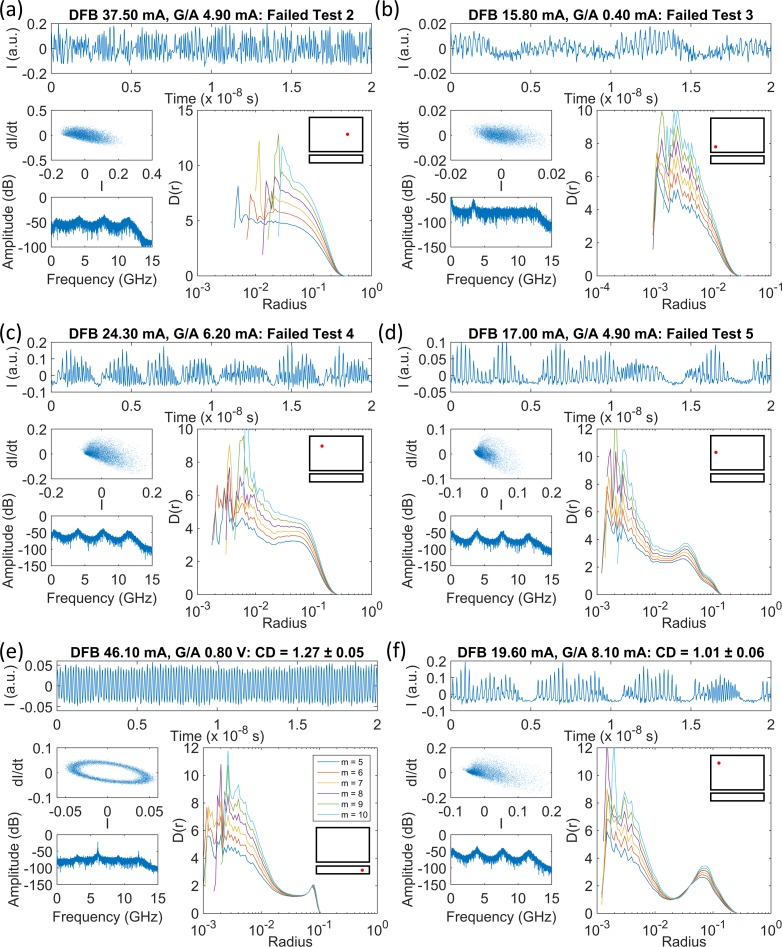
Partial output power time series, phase diagrams, FFT spectra and gradient versus hypersphere radius plots (*D(r)* versus *Radius*) for the PIC laser. DFB currents and G/A section currents/voltages for the four-section PIC laser are shown in each figure. The legend in (e) shows the embedding dimensions, *m*, associated with the colours of each gradient curve. Curves are shown for *m* = 5–10. The positions of each time series in the parameter space are shown schematically by the red dots in the insets to each *D(r)* versus *r* figure. No scaling regions were detected using the algorithm in (a)-(d). A CD of 1.27 ± 0.05 was measured using the minimum detection algorithm in the case of the periodic signal in (e). A narrow minimum is found in each gradient curve in (f), where the time series is from the feedback gain region. The tests referred to are described in Table 2. All curves increment by gradients of approximately 1 at short radii as *m* increases by 1. This occurs due to noise effects and finite sampling. Increments of 1 per embedding dimension at larger radii are indicative that the upper embedding dimension of 10 is insufficient to completely unfold the attractor, i.e. higher dimensional chaos.

The Grassberger-Proccacia algorithm outputs a set of correlation sum data for a range of hypersphere radii (effectively the number of pairs within a given point-to-point distance on the attractor). There is usually a variation in the gradients of the curves for each different embedding dimension, except for where a scaling region exists. When analysing a small number of time series, the gradient plots can be individually inspected for scaling regions. However, the data sets recorded from the three laser systems studied here contain tens of thousands of individual time series, so an automated detection procedure was necessary. Some automated procedures have been described elsewhere [10, 11, 15], although here we use a new algorithm, specifically designed to detect the type of scaling regions found in the gradient plots obtained for the low complexity time series from the PIC laser in the first instance. A full explanation of the algorithm, called the ‘minimum gradient detection algorithm’, is provided in Section B in S1 File, “A tutorial on finding scaling regions in gradient, D(r), versus radius plots using the minimum gradient detection algorithm”. A summary of the separate tests applied to the correlation sum gradient data for each time series is presented in Table 2. It references Fig 7 which shows examples of time series for the PIC laser system as examples using experimental time series with noise. These cases and the detailed implementation of the process are described in Section B in S1 File.

**Table 2:** Minimum gradient detection algorithm applied to correlation sum gradient plots.

Test Number	Minima gradient values as a function of hypersphere radius, in each embedding dimension plot, are tested for:	Description	Example Figures
1	Unsuccessful gradient calculation	This test checks whether the TISEAN programme has returned data for the gradients of the correlation sum for at least one hypersphere radius value.	No gradient data generated
2	Narrow radius range for largest embedding dimension	Excludes time series where the minimum within any of the lower embedding dimension gradient curves appears at a radius where there is no data to compare against in the curve for the highest dimension.	Fig 7(A)
3	Flatness	Minima are considered to be not flat enough if the points at radii to either side of a minimum are more than some threshold percentage higher than the gradient sum at the minimum. (Threshold set to 10% for the PIC and SLwOF, 40% for the OISSL system.)	Fig 7(B)
4	Radial separation	Minima found in different embedding dimensions occur at widely separated radii and therefore do not constitute a scaling region. (Minima found at radii that were more than 5 radius values away from the median radius of the minimum in each embedding dimension were considered invalid.)	Fig 7(C)
5	Large vertical spread	Minima in most gradient plots occur at the same radius for several embedding dimensions. However, where there is a large separation on the gradient axis, a true scaling region is not found.	Fig 7(D)
Robust CD Result	Small vertical spread	If the vertical spread between minima is small enough, then the average of the minima is considered to be a measurement of the CD. (Each minimum was required to be within ± 0.25 of the average gradient value for the minimum in each embedding dimension.)	Fig 7(E) and 7(F)

Consideration was given to whether noise filtering the time series before completing the CD analysis would lead to improved results. Trials indicated there may be a small advantage in identifying period 1 data when noise filtering is applied. But, noise filtering has a negative impact on obtaining robust CD values from chaotic data.

## 4. Results and discussion

### 4.1 Photonic integrated chip (PIC) laser

Fig 7 shows some time series from the PIC laser that were categorised by the minimum gradient detection algorithm, according to the tests outlined in Table 2. FFT spectra, phase plots and gradients plots (*D(r)* versus *r*) are provided in each figure in addition to a partial snapshot of the time series. Fig 7(A)–7(D) display gradient plots where no clear scaling region could be detected. The time series were excluded from the CD measurement algorithm at Tests 2–5, respectively. Fig 7(E) is one example of a time series for which a genuine scaling region was found and the algorithm returned a robust CD result. The time series is periodic, although there is some variation in the amplitude and some additional low-level noise. There is a flat scaling region across a small range of radii and a sensible CD value of 1.27 ± 0.05 was returned.

Fig 7(F) shows a time series with a series of regular pulse packages [32, 44]. The broad peaks at 3.9, 7.7 and 11.6 GHz in the FFT spectrum are harmonics of the external cavity frequency, while the peak at 0.3–0.5 GHz accords with the period of the amplitude dropouts in the time series. The gradient versus radius plots diverge at short radii for each increase in the embedding dimension, because of the smaller number of points in small hyperspheres, and, noise may disrupt the small-scale structure of the attractor. This is also observed in Fig 7(A)–7(E). At moderate radii, the curves all converge to a single gradient value. The curves then ‘kick up’ to higher gradients as the radius increases, before decreasing to 0 at radii beyond the largest point-to-point separation within the attractor. The point of most interest is the common minimum in the gradient curves for each embedding dimension at moderate radii, at an average gradient of 1.01. It could be a very narrow scaling region, with a value equal to the CD. However, these types of gradient curves are also potentially misleading. The same type of regular pulse packages are found in neighbouring parts of the parameter space, but the average gradients are higher. It may be that in those cases noise may be concealing a scaling region at a lower gradient, as reported in [6]. The gradient at the minimum should therefore be interpreted as an upper limit on the CD of the system.

**Fig 8:**
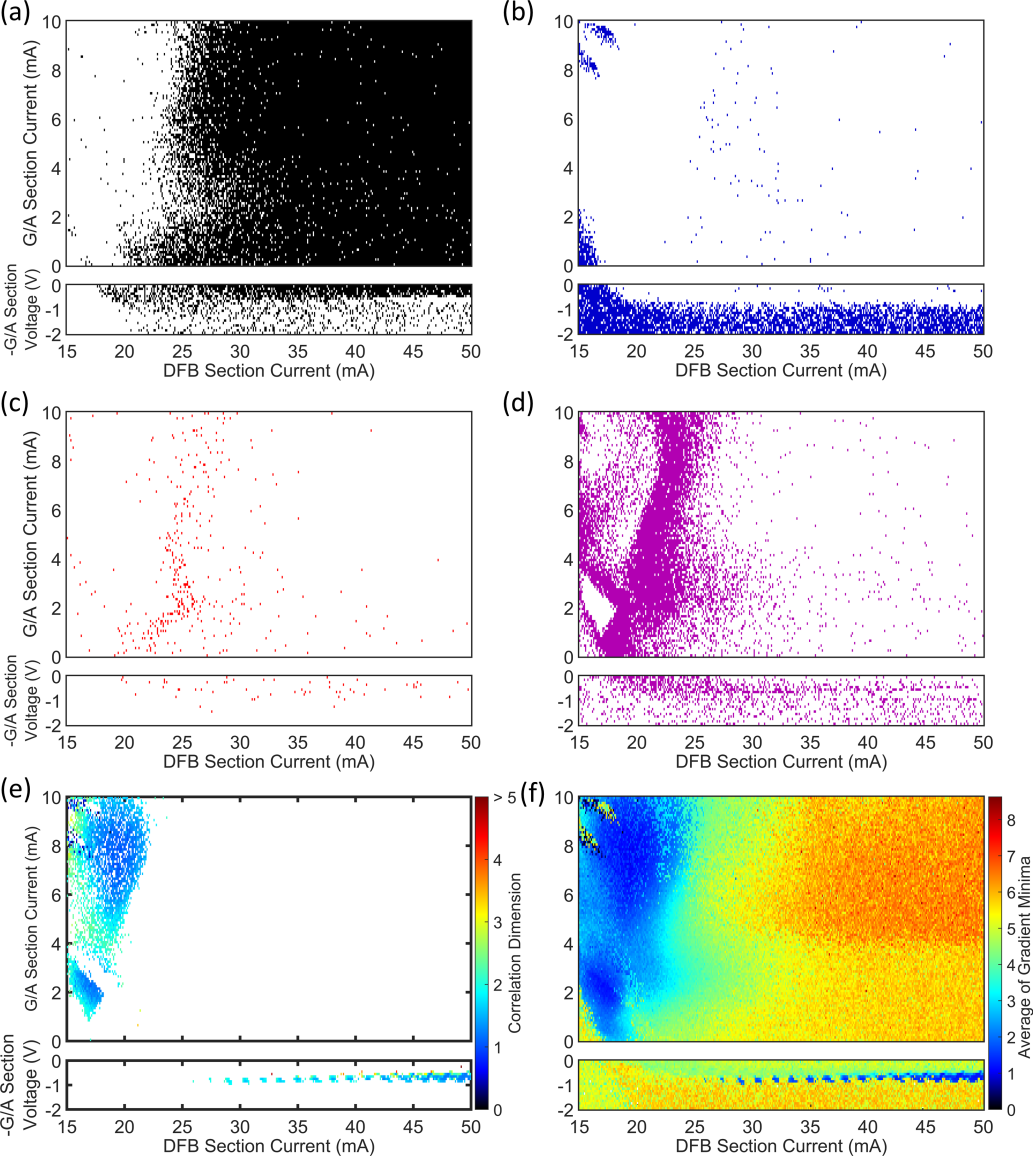
Maps of the minimum gradient detection algorithm test results for the PIC laser. (a) Test 2, (b) Test 3, (c) Test 4, (d) Test 5. (e) Scaling regions were detected for a small part of the map at DFB currents below 22 mA when feedback was enhanced, and at reverse feedback section biases of 0.6 to 1 V. (f) A map of averaged *D*_*m*_*(r)* curve minima, prior to any testing. Those regions of the parameter space where scaling regions were not detected generally have high values for the average of minima.

The 42 822 time series recorded from the PIC laser were analysed automatically using the minimum gradient detection algorithm. Fig 8(A)–8(E) map the time series according to the outcome of the algorithm at the various test stages. Fig 8(A) maps those time series for which the algorithm failed to progress beyond Test 2. Most of the time series at DFB section biases above 25 mA–with gain applied to the feedback section–are present in this map. This outcome is consistent with the expected behaviour of a Type B laser perturbed by optical feedback, i.e. the laser readily delivers chaotic outputs. It also appears that most of the time series corresponding to reverse voltages from 0 to approximately 0.6 V on the feedback section fail Test 2 and can also be classified as chaotic. This indicates that reverse voltages of more than approximately 0.6 V are necessary to sufficiently attenuate the light from the external cavity to prevent coherence collapse. The map in Fig 8(A) can be interpreted as showing the region of higher dimension dynamics with CD beyond 5 that cannot be robustly determined with the present specifications of time series length.

Fig 8(B) is a map of those time series where the minimum gradient detection algorithm did not proceed beyond Test 3. The minima found in the gradient plots for several embedding dimensions were not flat and long enough to constitute part of a scaling region. The highest concentration of points in the map is for reverse feedback section voltages of 1 to 2 V, where the laser is operating essentially without optical feedback, i.e. free-running conditions. The laser is also below the threshold injection current at all feedback section voltages for a small part of the parameter space where the DFB section bias is below 18 mA, i.e. there is no output from the laser. The laser operated above the threshold current at all points mapped for positive feedback currents. At gain section biases of 0–2 mA and DFB section biases of 15–17 mA, the laser output power is weak. The time series amplitudes are low and the FFT spectra reveal only a weak peak at the first harmonic of the external cavity frequency (see Fig 7(B)). At DFB section biases of 15–18 mA and high gain section biases of 7.8 to 10 mA, the laser switches on small time scales between CW operation and output with regular pulse packages [32]. In all cases the laser is operating in a stable or low-complexity state, where the noise in the system is comparable to or larger than any dynamical behaviour from the laser. Fig 8(A) and 8(B) provide a striking illustration of how effectively the different dynamical states of the laser can be isolated and mapped by a relatively simple automated analysis of the features within the gradient versus radius plots. This observation is explored further in section 4.4 of this paper.

Fig 8(C) and 8(D) map parts of the parameter space where there is no clear scaling region in the gradient plots, but where the time series are likely to be less complex than those mapped in Fig 8(A). Minima were found where the gradient plots were acceptably flat, but occurred at widely spaced radii (Fig 8(C)) or where the gradients at the minima were spread broadly along the vertical axis for different embedding dimensions (Fig 8(D)). Interpreting the associated gradient plots (Fig 7(C) and 7(D)) as displaying ‘moderate complexity’ is consistent with the fact that these maps are most populated at the lower end of DFB injection currents in the feedback gain biased region. Under a reverse voltage bias on the feedback section, the data points extend across the DFB current range, but the highest concentration remains at low currents.

Fig 8(E) maps the time series where ‘robust CD measurements’ were obtained. As such, the data points are coloured according to the CD assigned by the minimum gradient detection algorithm. The map reveals that the CDs of the laser output time series can only be defined with some confidence in a small fraction of the operating parameter space. At reverse voltages of 0.6 to 0.9 V on the feedback section (absorption), the laser output is periodic, as in Fig 7(E). The CDs of the time series vary in the range from slightly higher than 1 to approximately 2.5. Periodicity in the output appears and disappears as the DFB section current increases. The dynamics are quite different where a gain bias is applied to the feedback section. For DFB section biases below 22 mA, a wide range of gain feedback biases deliver a low-complexity output. Inspection shows that these time series are in the form of Fig 7(F), with regular pulse packages at the external cavity frequency [32]. The gradient plots are also like those found in Fig 7(F), where there is a distinct set of minima at a common radius in all embedding dimensions. As discussed earlier, the gradient value at these minima which range from 1 to 3.4 may be a true reflection of the CD for the system, but the values may be inflated due to noise and they are best interpreted as an upper limit on the CD for the time series.

Robust CD measurements were obtained for the part of the laser operating parameter range shown in Fig 8(E). However, an indication of complexity elsewhere in the map can be inferred from a simplification of the minimum detection algorithm. Instead of classifying gradient plots according to the tests in Table 2, the lowest local minima found at each embedding dimension in the plots can be averaged. Now, since no tests are applied there is no way to know that the gradient minima are from scaling regions, but as Fig 8(F) shows, this simple procedure remains informative. The map does not show true CD values, but does reflect the earlier observations that there are two main regions of the map (blue) with low complexity. Additionally, the average of the gradient minima increases as the gain on the feedback section and DFB section current increase, indicating a transition to chaos. The apparent systematic increase in these pseudo-CD vales is also consistent with the systematic increase observed from permutation entropy mapping (Fig 2 and [34]). The average of the minima is also high for feedback section reverse voltages of 1 to 2 V, where the laser output is CW and the noise on the signal is the dominant dynamical feature.

### 4.2 Semiconductor laser with optical feedback (SLwOF)

**Fig 9:**
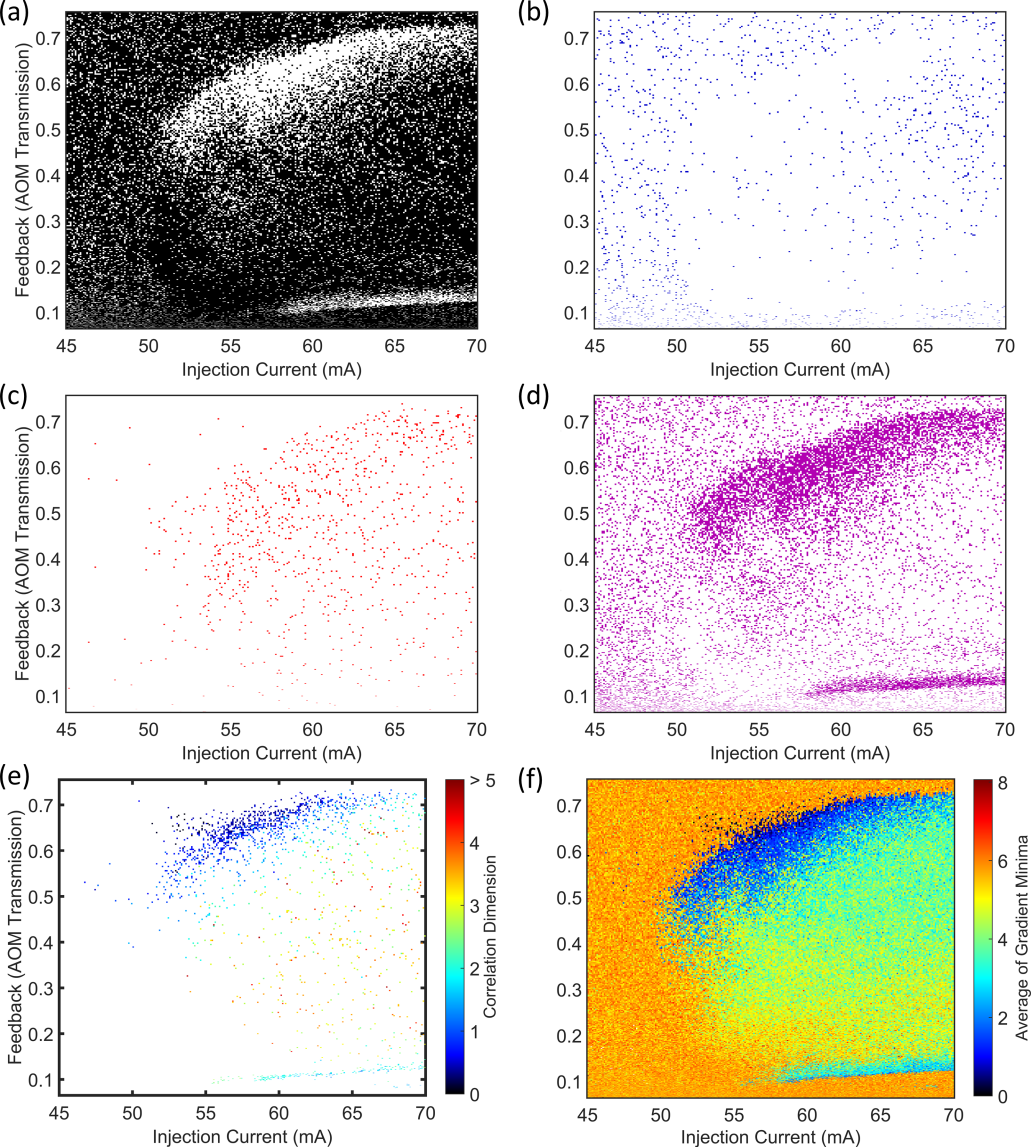
Maps of the minimum gradient detection algorithm test results for the SLwOF system. (a) Test 2, (b) Test 3, (c) Test 4, (d) Test 5. (e) Scaling regions were detected in two main bands at high and low fractions of optical feedback. (f) Average of minima without testing.

After applying the minimum gradient detection algorithm to map the laser power output time series for the SLwOF, we were able to learn more about the complexity of the laser than was possible in prior studies. Few time series failed Test 1, so a figure is not supplied for this result. Fig 9(A) shows that the majority of the time series in the laser parameter space fail to return a measurement of the CD. For these time series the gradient plot for the highest embedding dimension was too narrow, thus there was a failure at Test 2. This is indicative of the CD associated with the time series being higher than the range interrogated with the time series specification. The exception to this is found in the two white coloured bands at injection currents above 50 mA. The first band is found at AOM transmission fractions of approximately 0.1 to 0.15 and the second is found at transmission fractions of 0.45 to 0.7. In the lower band, the laser transitions from CW operation to low frequency fluctuations and then coherence collapse as the feedback level increases. The opposite occurs with increasing feedback level in the upper band where the laser becomes less chaotic before the sharp transition to constant output power [30].

In the PIC laser system, there was a clear distinction between the parameter spaces mapped by Tests 2 and Test 3 in Fig 8(A) and 8(B). For the SLwOF system, we don’t observe the same strong delineation between the test results in Fig 9(A) and 9(B). The reason for this difference is discussed in section 4.4, but is due to the discrete nature of the time series amplitude measurement at small detection voltages in the PIC laser setup, which was not the case for the SLwOF.

Fig 9(C) shows that the time series failing Test 4 are distributed widely in the parameter space. They are concentrated most heavily at injection currents higher than 50 mA, i.e. in the coherence collapse region, but also through the upper white band from Fig 9(A). There are very few points mapped in those parts of the parameter space where the RMS amplitudes of the time series are low [30]. Fig 9(D) is almost a negative image of Fig 9(A), with most points mapped fitting into two bands at AOM transmission fractions of approximately 0.1 to 0.15 and at fractions of 0.45 to 0.7. These bands are found to contain time series with regular, spiky dynamics. The algorithm successfully categorises them, but clearly did not detect any scaling regions.

Fig 9(E) shows those time series for which a robust measurement of the CD was made. Values between 0.04 and 5 are found. In agreement with previous research these points are mostly close to the lower and upper transition boundaries between CW and unstable dynamics [31]. In the lower band at AOM transmissions of 0.1 to 0.12, the CD values measured lie generally in the range from 1.7 to 2.3. Many more robust results are returned in region near the upper boundary i.e. the higher AOM transmission band. A wider range of CD values, from 0.1 up to approximately 2.5 is found in this region. Additionally, it appears that there is a trend towards increased CD values in this band, as the injection current increases and the AOM transmission reduces. This spreads into a smattering of high CD values (1–5) in through the coherence collapse region.

Fig 9(F) once again shows the average of the gradient minima found in each embedding dimension within the gradient plots, where no testing was applied. The two bands of low complexity at low and high optical feedback (AOM Transmission) contrast distinctly with the CW region, which is coloured predominantly orange, with averages in the minima at approximately 5 to 6. The averages in the coherence collapse regime between the two bands and at injection currents above 50 mA are in the middle range of the scale, at approximately 3 to 5. This is consistent with there being some structure in the dynamics that is less complex than the noise that elsewhere overlays the CW dynamics.

**Fig 10:**
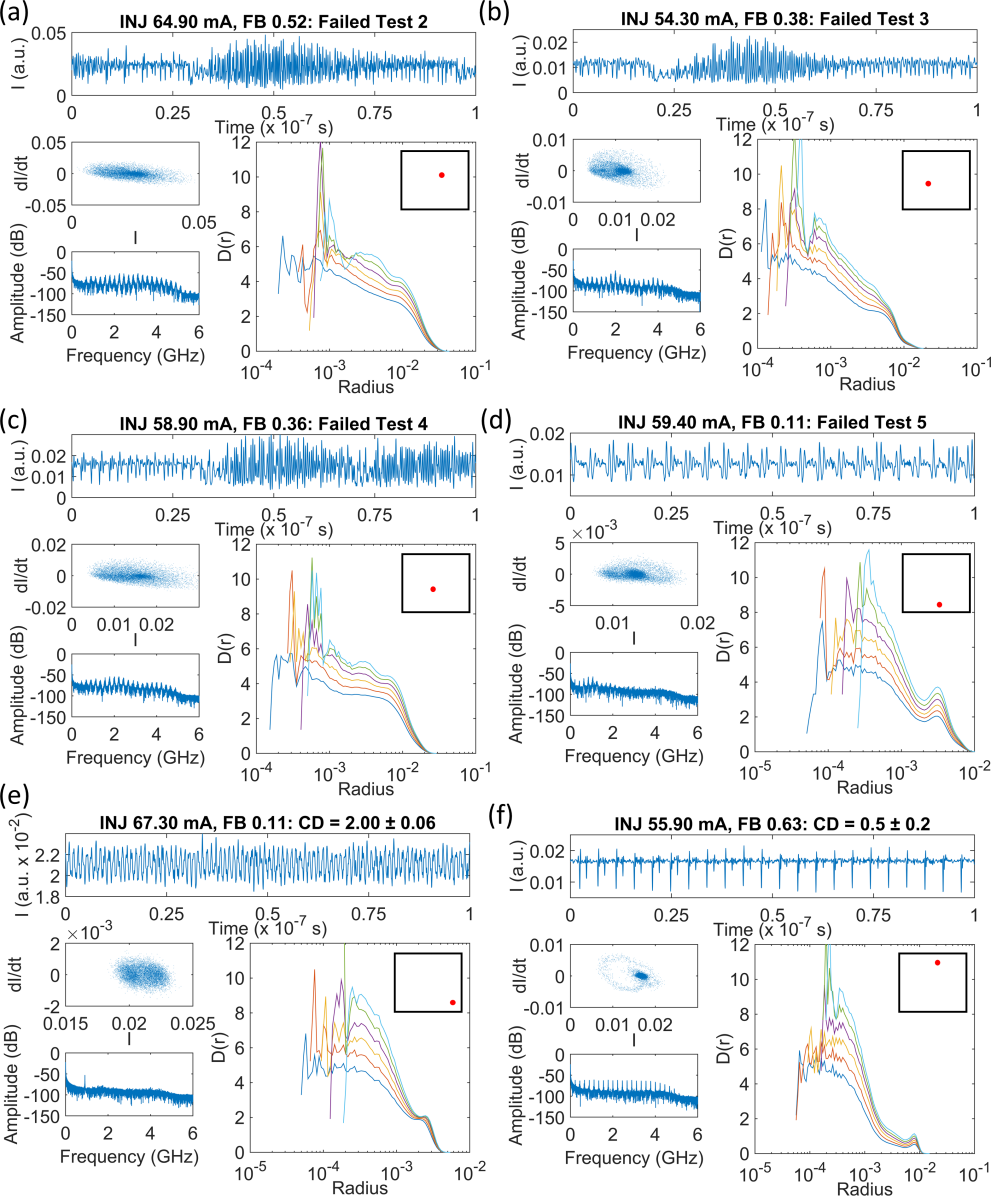
Time series, phase portraits, FFT spectra and gradient versus radius curves for selected SLwOF data. The time series fails the following tests: (a) Test 2, (b) Test 3, (c) Test 4, (d) Test 5. The relative positions of the time series in the laser parameter space are shown schematically by the red dots in the insets to each *D(r)* versus *r* figure. (e) A time series with a narrow scaling region and CD = 2. The main difference between the gradient curves presented here and those from the PIC laser is for the result of Test 3; compare Fig 7(B) and Fig 10(B). In Fig 7(B) the gradient curves for all embedding dimensions have the same radius range and are spiky across the full radius range. In Fig 10(B), the curves are mostly smooth at intermediate radii and long radii, but there is a deep spike at a single radius for some of the curves.

In Fig 10, time series, phase portraits, FFT spectra and gradient curves are plotted for representative data points from the maps in Fig 9. Fig 10(A)–10(D) do not show any scaling regions, as expected. Fig 10(D) is similar to Fig 7(D), where it appears that the gradient plots are converging towards, but not yet reaching, a common minimum at a moderate radius value. Fig 10(E) shows a time series from the lower band of ‘robust’ CD values. This has a single narrow peak in the FFT spectrum and the time series appears to be periodic, but has an irregular amplitude. The CD of 2 appears to be a sensible measurement. On the other hand, Fig 10(F) shows a time series from the upper band of chaotic CD values. The average minimum gradient value returned was 0.5 ± 0.2. The time series appears to be spiky, the phase portrait shows a large diameter loop and the FFT spectrum indicates the presence of 23 harmonics of the external cavity round trip frequency. There may be even more harmonics that weren’t measured because the oscilloscope bandwidth was limited to 4 GHz. The minimum in each gradient curve is clearly identified, but the value of 0.5 ± 0.2 is not consistent with the CD of 1 expected for a system dominated by multiple, commensurate harmonics. This low CD value appears to be a systematic signature of the presence of several harmonics. This feature will be discussed further in a future work.

### 4.3 Optically injected solid state laser (OISSL)

**Fig 11:**
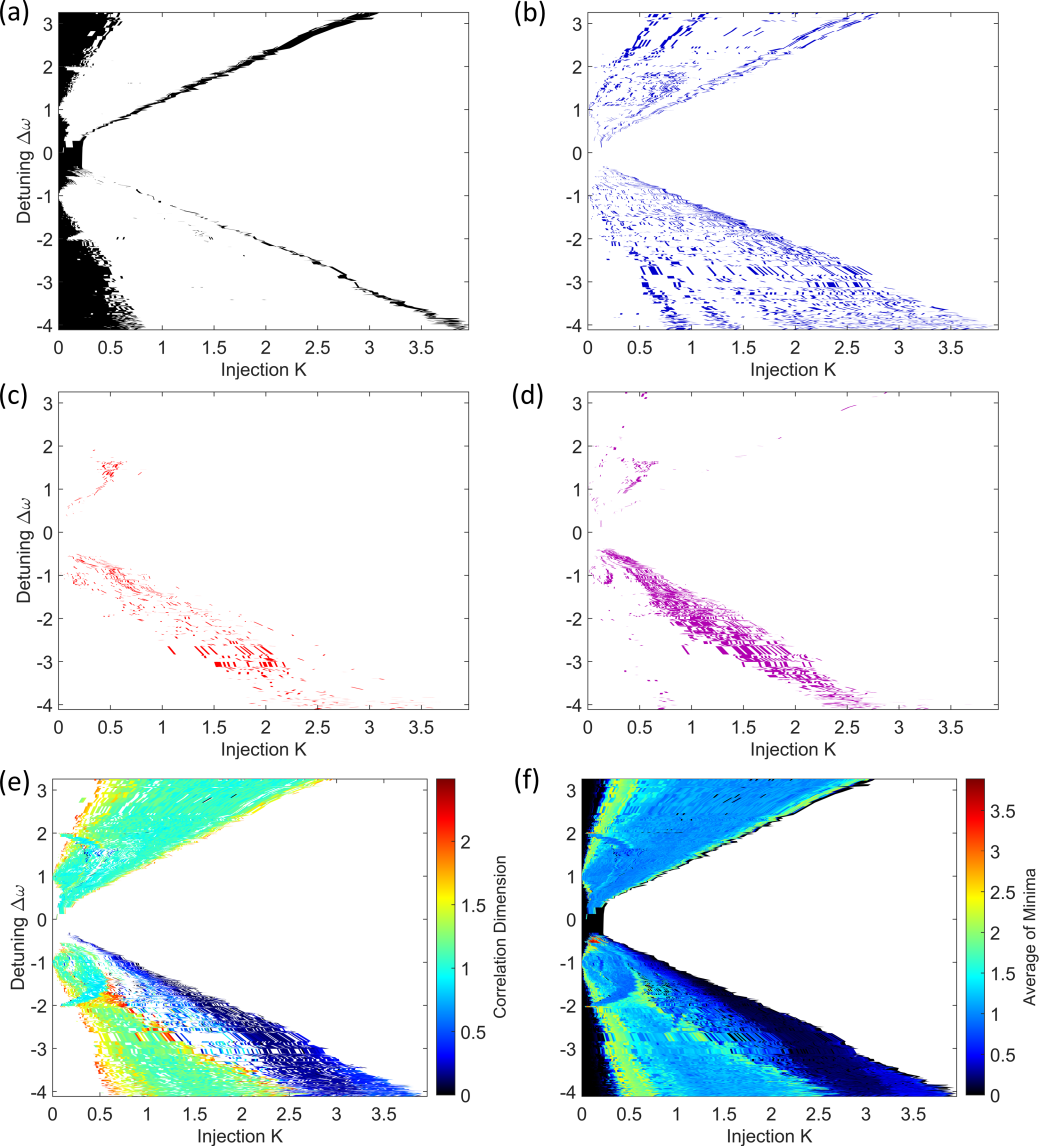
Maps of the minimum gradient detection algorithm test results for the OISSL system. (a) Test 2, (b) Test 3, (c) Test 4, (d) Test 5. (e) Shows that a robust scaling region was detected in the majority of the parameter space for which calculations were made. (f) Shows the average of minima without testing. The white area on all the maps is the injection locked region for which no time series were recorded in the experiment. Such time series would be characterised by the noise on a CW output power and would fail Test 2 for that reason had they been recorded.

Maps of the results from the minimum gradient detection algorithm for the OISSL system are presented in Fig 11. Fig 11(A) shows that those time series at low injections strengths–from 0 to 0.5 –tend to fail Test 2. Additionally, there are two lines which stretch across the injection strength range where the system transitions to the injection-locked region [38, 40].

**Fig 12:**
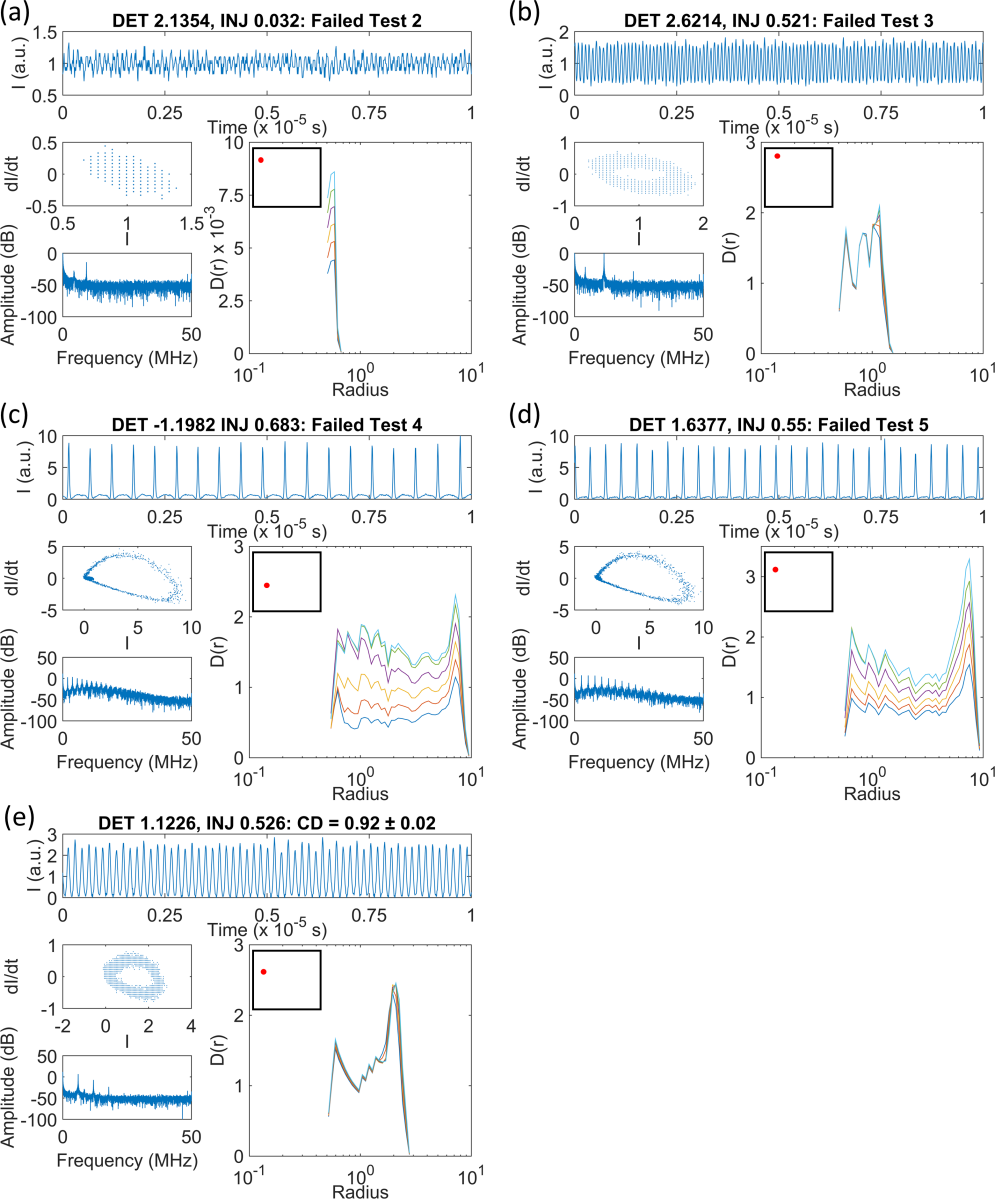
Time series, phase portraits, FFT spectra and *D*_*m*_*(r)* versus radius curves for some OISSL data. (a) Test 2, (b) Test 3, (c) Test 4, (d) Test 5, (e) a time series with CD = 0.92 ± 0.02. The time series is periodic with an irregular amplitude and the phase portrait has the form of a broad limit cycle. The CD value near 1 is consistent with a periodic time series, however the gradient curves do not contain a scaling region across a wide range of radii. The relative positions of the time series in the laser parameter space are shown schematically by the red dots in the insets to each *D(r)* versus *r* figure.

Fig 11(B) shows those time series which failed Test 3. The parameter space of the mapped time series does not overlap with the parameter space for the results of Test 2. This is similar to the case in the PIC laser system. The threshold for the flatness of the gradient minima in Test 3 was relaxed when analysing this system. The points adjacent to the gradient minima in each embedding dimension were allowed to be 40% higher than the value of the minimum. For the other two lasers, a difference of only 10% was allowed. The test was adjusted because there are a number of points in the map where the laser output is mostly periodic, but the gradient plots do not display flat, wide scaling regions (see Fig 12(E)). The test was relaxed to appropriately map these regions of periodic output, noticeably in two crescent shaped regions at small injection strength and detunings in the range -2 to 2, which correspond with period doubling from the bifurcation diagram [40].

Fig 11(C) and 11(D) show that most of the time series with incompletely formed scaling regions are present at negative detunings, across a range of injections from 0 to 3. Fig 11(E) reveals that acceptable measurements of the CD are obtained for the majority of the parameter space. In fact, there are many more ‘robust CD results’ for this laser compared to the PIC and SLwOF. This is because the OISSL system does not operate with a highly complex chaotic output in most of the parameter space. At positive and negative detunings, CD values of 1 to 2 are frequently measured. CD values above 2 are only found within thin strips at the bifurcation boundaries. These are adjacent and intermixed with time series that fail Test 2 as seen by comparing Fig 11(A) and 11(E). Additionally, as observed in the SLwOF, and previously noted for this system [15] CD values between 0 and 1 were found in a large part of the parameter space. As can be seen from Fig 12, the laser delivers pulsed outputs for much of the parameter space and in certain cases the minimum gradient detection algorithm associates very small CD values with this behaviour. This does not accord with the normal interpretation of CDs in periodic time series and is a point that will be covered in a separate paper.

### 4.4 Impact of discretisation of signal values near the detection noise level

**Fig 13:**
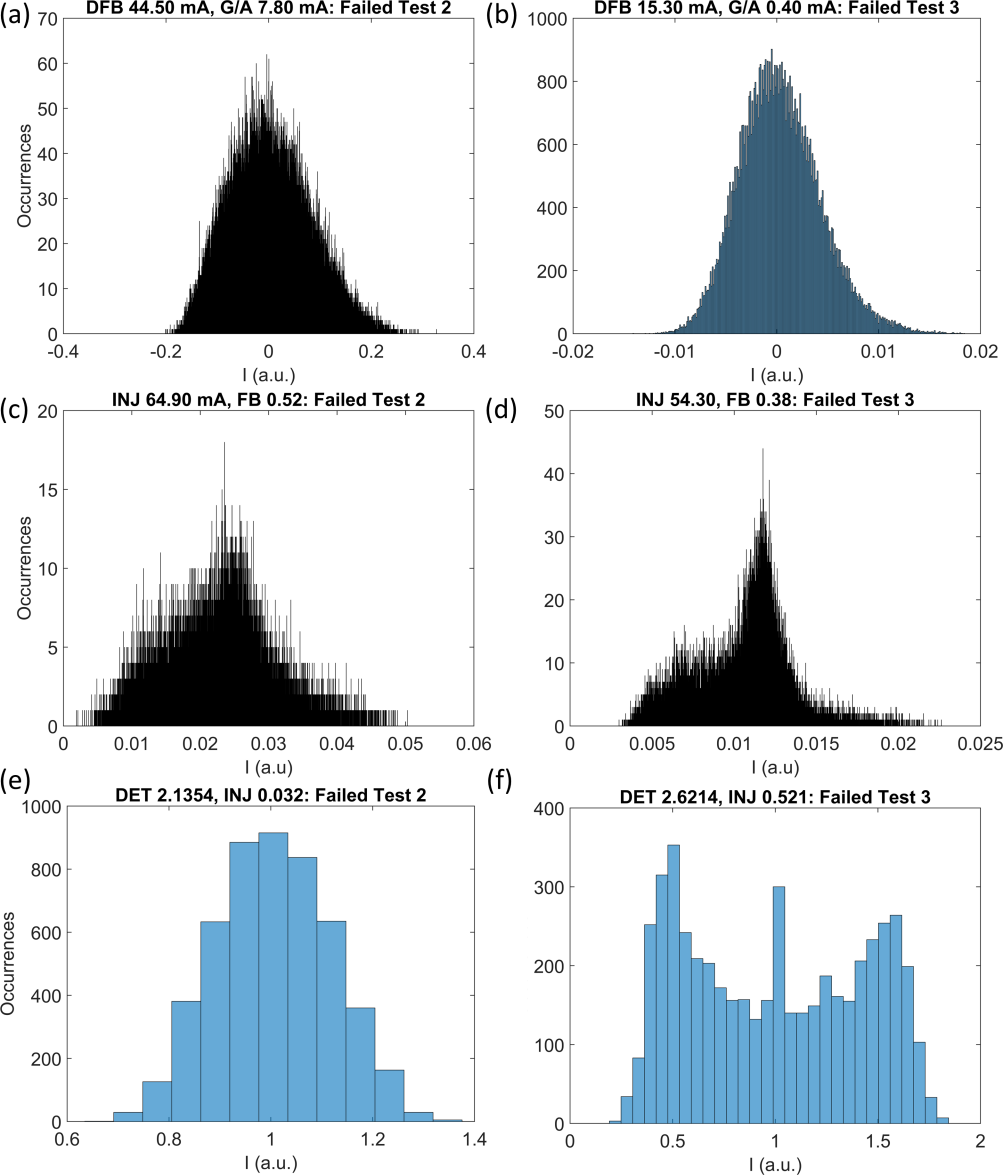
Histograms of time series failing tests 2 and 3 in the three laser systems. PIC laser (a) and (b), SLwOF (c) and (d) and the OISSL system (e) and (f). The number of points in the time series are 80 000, 20 000 and 5000 for the PIC, SLwOF and OISSL systems, respectively. The voltage/div on the oscilloscopes used as a measure the power output from the PIC and OISSL systems was larger than in the SLwOF system, so that the full range of excursions for highly complex outputs could be measured. This meant that in these two laser systems the low amplitude fundamental noise output in other parts of the parameter space was coarsely sampled. The detected power values for ‘noisy’ time series are spread into fewer bins in the PIC laser and OISSL histograms than in the SLwOF histograms.

**Fig 14:**
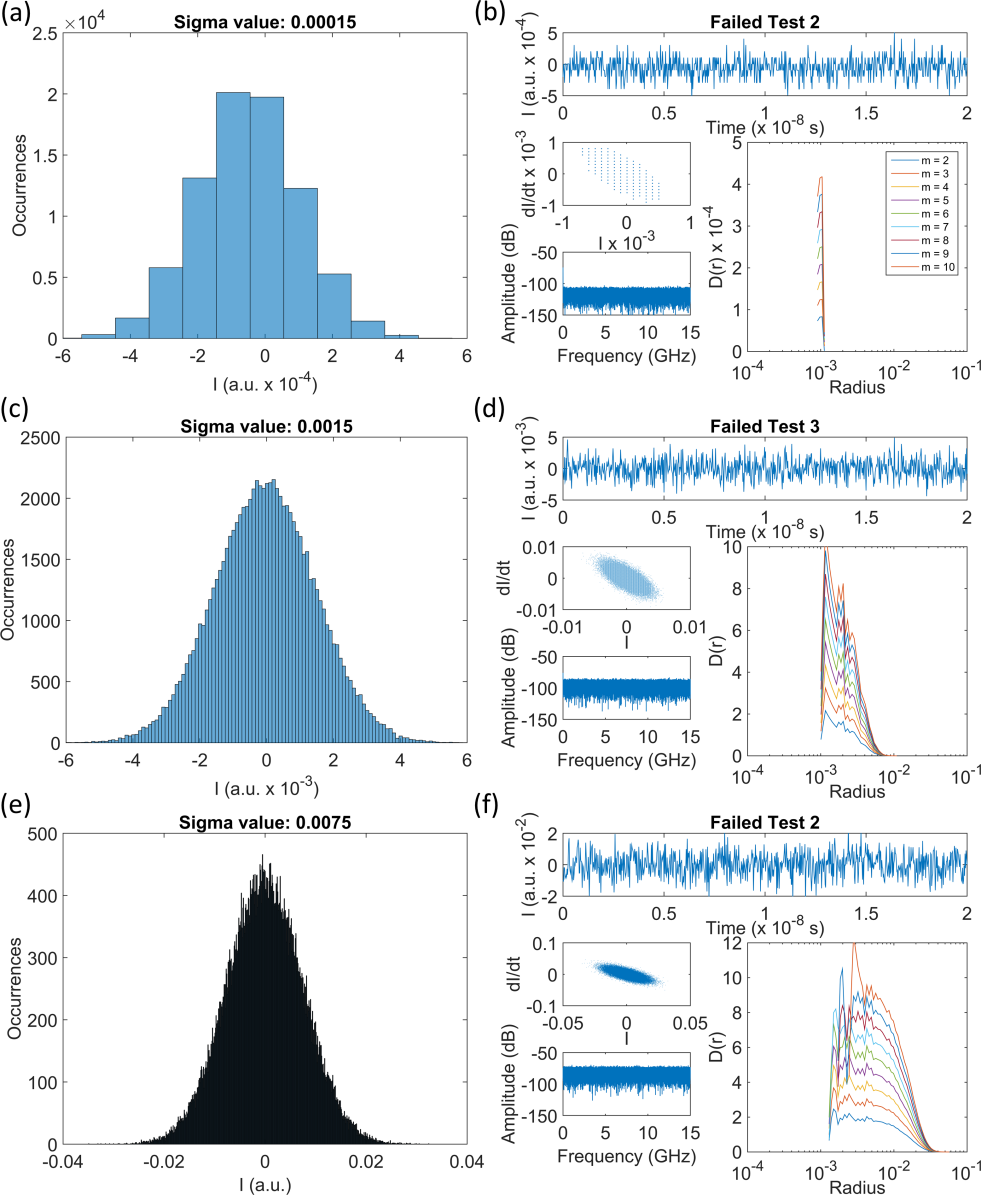
Results of the minimum gradient detection algorithm for synthetic timeseries. Histograms of synthetic time series, generated using a normalised Gaussian distribution (a, c, e) and the corresponding time series, phase portraits, FFT spectra and *D*_*m*_*(r)* versus radius curves. Time series consist of 80 000 points, with standard deviations of (a—b) 0.00015, (c—d) 0.0015, (e—f) 0.0075. The test results were obtained using the minimum gradient detection algorithm.

One of the major differences in the analysis of the three laser systems using the minimum gradient detection algorithm is the classification of the time series into those failing Tests 2 and 3. In the PIC laser maps (Fig 8(A) and 8(B)) and in the OISSL system maps (Fig 11(A) and 11(B)) the regions of the respective parameter spaces represented by either Test 2 or Test 3 are spatially separated. On the other hand, in the maps of the SLwOF system (Fig 9(A) and 9(B)), the Test 2 and 3 failures are mapped in an overlapping region of the parameter space. In the PIC and OISSL systems, the minimum voltage steps of the digital oscilloscopes used to measure the laser outputs were similar to the magnitude of the fundamental and technical noise. This was a direct consequence of setting the overall scale to collect the full amplitude of the output power associated with the largest excursions throughout the mapping. In the SLwOF system the maximum power excursions were much closer in value to the excursions associated with the system noise. Thus the noise covers a large number of the discrete voltages associated with the digitisation of the signal. The effect that this has on the distribution of amplitude values in the recorded time series and the shapes of the gradient versus radius curves are illustrated in Figs 13 and 14 and explained next.

Histograms of the recorded power in selected time series from the PIC, SLwOF and OISSL systems are shown in Fig 13. Fig 13(A), 13(C) and 13(E) represent histograms from time series failing Test 2 and Fig 13(B), 13(D) and 13(F) correspond to time series failing Test 3. The histograms for the PIC laser, Fig 13(A) and 13(B), appear to be Gaussians with a slight skew to the high voltage end. The bin widths are the same in each histogram, but the power range in Fig 13(B) is much narrower than in Fig 13(A), ~0.01 compared with ~0.25. There are many more occurrences of low power values in the time series failing Test 3, as compared with the time series failing Test 2. This contrasts with the histograms for the SLwOF system in Fig 13(C) and 13(D), where the values of power measured in both time series span a wide range of discrete values, ~0.05 and ~0.025, respectively. The peak number of occurrences within the bins of each histogram does not differ greatly in either case. The histograms of the OISSL system, in Fig 13(E) and 13(F), have more in common with those from the PIC system, where only a small number of discrete power values are spanned. The contrast with the PIC laser is that fewer power values are spanned by the time series failing Test 2 than the time series failing Test 3. Further analysis of these observations is provided with reference to Fig 14.

Fig 14 shows power value histograms and gradient versus radius plots for three time series that were generated with the form of normalised Gaussian distributions. The power values were discrete rather than continuous, with an amplitude resolution equivalent to that of the oscilloscope used for the measurements of the PIC laser data. The sampling interval and number of points was set to be identical to that of the PIC laser experimental time series, while the standard deviation of the distributions was set to 0.00015, 0.0015 and 0.0075, respectively. The number of points in the central bin of the histograms decreases as the standard deviations of the time series increase. Fig 14(A) is similar to the histogram for the OISSL system in Fig 13(E), in that only around 10 discrete power values are spanned by the histogram. Fig 14(C) shows a distribution similar to the histogram for the PIC laser in Fig 13(B). The time series clearly spans a wider range of discrete power values, but there are still thousands of occurrences of the values near 0. Fig 14(E) shows that once again the histogram spans a wider range of discrete power values as the standard deviation increases. With an even greater standard deviation, the distribution would tend towards that plotted in Fig 13(A).

The three time series, phase portraits, FFT spectra and gradient versus radius plots are shown in Fig 14(B), 14(D) and 14(F). The phase portraits and FFT spectra are similar in all cases and are consistent with white noise. The gradient plot in Fig 14(B) shows that gradients were only calculated for three radius values in the time series with the smallest standard deviation, and there are no local minima (turning points) in the plot. The time series from the OISSL system that were mapped in Fig 11(A) had similarly narrow gradient plots. Results of this type were classified as having failed Test 2 of the algorithm.

The gradient curves in Fig 14(D) for the Gaussian noise time series with the second highest standard deviation have the ‘spiky’ appearance familiar from Fig 7(B). Peaks and troughs occur at the same radii in all embedding dimensions and the magnitudes of the spikes lead to a classification failing Test 3 in the minimum gradient detection algorithm. This analysis shows that it is the discretisation of the oscilloscope at the noise level for the laser system that leads to the characteristic appearance of these particular gradient curves. The points in the time series appear in a small number of discrete amplitude levels. When an attractor is formed using the method of delays and the correlation sum is calculated, point-to-point pairs are counted within a hypersphere of steadily increasing radius. However, there are only a small number of point-to-point pairs at certain radii because of the clustering of points in the attractor. Thus, the correlation sum gradient increases and decreases sharply as the hypersphere radius increases since the distribution of points within the hypersphere is non-continuous.

Fig 14(E) and 14(F) show that as the standard deviation of noise increases within the time series, the minimum detection algorithm reports a failure of Test 2, instead of Test 3. With an increasing standard deviation, the points are distributed more continuously through a larger range of amplitudes. This means that the attractors formed by the method of delays have a more continuous distribution of points through a hypersphere of increasing radius. The gradient of the correlation sum varies more smoothly through neighbouring hypersphere radii, particularly at the moderate to large radii. Additionally, Fig 14(F) shows a reduction in the width covered of the gradient curves as the embedding dimensions increase. This was exactly the type of behaviour observed in the analysis of experimentally measured time series like that in Fig 7(A).

In summary, when the distribution of points in a noisy time series is too narrow, the gradient curves will also be constrained to a narrow range of radii and the minimum gradient detection algorithm will deliver a failure at Test 2. If the distribution of points increases, consistent with a range of small standard deviations, the gradient versus radius curves have a spiky appearance resulting in failure at Test 3. With greater standard deviation, the time series become more continuous and the test result will be a failure of Test 2 commensurate with noise/high complexity of a large enough amplitude to populate a sufficient range of discrete measurement levels. Thus, when the measurement of the output of a laser or any other system is discrete and the output amplitudes are similar in scale to the discrete steps recorded by the measurement apparatus, time series failing Tests 2 and 3 will be mapped in a distinct manner as in the PIC and OISSL laser data. Where the sensitivity of the measurement system is much greater (the power/division is smaller) and there is a wide distribution of points into different power values, as in the SLwOF data, the spatial separation in the maps of the Test 2 and Test 3 results is less distinct. This approach may be able to be deliberately deployed to differentiate fundamental and technical noise from time series with high levels of complexity as has been seen for the PIC and OISSL data in this study.

## 5. Conclusions

To evaluate the suitability of a certain laser for a given physical application it is clearly desirable to produce detailed maps of different complexity measures, such as the CD and/or PE. The CD is favoured if it can be determined because it gives more information about the nature of the dynamics and more clearly differentiates variations at higher dimensions. Widely varying dynamical outputs, of a chaotic, pulsed, periodic or CW nature can be produced by a single, experimental laser system as the result of changes in the operating parameters. This can include dramatic changes for small changes in the operating conditions. It has been shown that these dynamical forms can be determined and differentiated by CD mapping in some cases. The minimum gradient detection algorithm for CD measurement examined in this work has provided useful insights into the dynamical outputs of three different laser systems. Several intermediate tests have been introduced in the algorithm and applied on the way to ultimately determining whether a scaling region is found in the gradient versus radius curves for the laser output time series. In the maps that result, which capture the sequential failure of the tests, as well as the final robust CD values, we have been able to objectively identify those parts of the operating parameter space where the output from a given laser system is characterised by dynamics such as fundamental/technical noise or chaos with dynamical noise. Those parts of the parameter space where scaling regions exist are clearly identified, but we have additionally mapped those areas where scaling regions have not fully formed. Nevertheless, the trend towards their formation informs that there is a lower level of dynamical complexity than in other parts of the parameter space.

The ability to discriminate between regions of output with fundamental/technical noise and those regions of chaos/dynamical noise in the systems studied here was due to a fortuitous selection of the measurement resolution. In the PIC laser and the OISSL system, the digital resolution was on a level commensurate to the amplitude of the fundamental noise. These outputs were therefore coarsely sampled and the resulting time series consisted of relatively few amplitude values. The analysis of some discrete, synthetic Gaussian noise distributions, with different standard deviations but constant amplitude resolution demonstrated that the gradient versus radius plots have a characteristic spiky form for small values of standard deviation. These curves are observed in the experimental systems where the output is known to be dominated by fundamental but discretised noise. In the SLwOF system, the sampling resolution of the oscilloscope was much smaller. The dynamically and/or noise varied output power was therefore well sampled. Thus, in this case the fundamental noise could not be easily distinguished from the large amplitude chaos. It can therefore be an advantage when interrogating the complexity of a nonlinear system to set the resolution of the measurement apparatus to a level at which the fundamental/technical noise is coarsely sampled, particularly where the signal to noise ratio is high in the other areas of the parameter space.

Producing high resolution maps of the CD value, and the related test failure maps, across a broad parameter range, for three different laser systems, has led to a significant increase in confidence that the CD values obtained are physically meaningful. Individual calculations of CD do not support such confidence and thus we commend this approach, as and when it is possible to use it in experimental settings, across different areas of nonlinear science. In most cases we did not tend to observe integer values of CD because of uncertainties introduced by the spread in the gradient versus radius curves for different embedding dimensions. However, we have been able to identify areas within the parameter space where the data is, for example, close to being periodic. Also, it is clear that some of the data shows trends towards the formation of scaling regions that nevertheless do not fully develop within the range of embedding dimensions used here. It may be worthwhile to further interrogate this data with other chaos analysis techniques.

## Supporting information

S1 File Calculating correlation dimension. Section A. Calculating the correlation dimension of a time series using the Grassberger-Proccacia algorithm. Section B. A tutorial on finding scaling regions in gradient, D(r), versus radius plots using the minimum gradient detection algorithm.
(PDF)
