# Supplementary material for: Insights on correlation dimension from dynamics mapping of three experimental nonlinear laser systems
Source: PLoS One. 2017 Aug 24;12(8):e0181559. doi: 10.1371/journal.pone.0181559 (PMC5570314; doi:10.1371/journal.pone.0181559)
Supplement: S1 File — Section A. Calculating the correlation dimension of a time series using the Grassberger-Proccacia algorithm. Section B. A tutorial on finding scaling regions in gradient, D(r), versus radius plots using the minimum gradient detection algorithm. (PDF) [file pone.0181559.s001.pdf]

S1 File. Calculating correlation dimension

S1.A Calculating the correlation dimension of a time series using the Grassberger-Proccacia algorithm

The dynamics of nonlinear systems can be visualised through plotting a phase space attractor, with each point on the trajectory of the attractor being described by a unique combination of system variables [1]. The number of axes for the plot must be equal to the number of system variables to adequately display the trajectory. For example, if a system evolves according to three time dependent variables, then a three dimensional plot of all the solutions to the system equations in a given time interval should reveal the geometrical form of the system attractor. A simple system, for example a periodic signal, is dependent on a single variable and is described by a line in the phase space. Lines have a geometrical dimension of 1. A quasi-periodic system, one in which the dynamics vary according to two independent periodic influences describes the surface of a torus in the phase space. This surface has a geometrical dimension of 2. Fractal attractors, which describe chaotic systems, are those with non-integer dimensions. The widely known Lorenz attractor has a fractal dimension of approximately 2.05, because the trajectory through the phase space is part way between a two-dimensional surface and a three-dimensional volume [1, 2].

Unfortunately, when dealing with nonlinear systems under experimental conditions, it is often difficult to measure a time series for more than a single system variable. For instance, in the work on the three lasers presented in this paper, it was only practical to measure the fluctuations in the laser output power with respect to time. Even if measurements of the time dependence of all system variables have not been measured, it is still possible to replicate the 'true' phase space attractor with a 'reconstructed' attractor using Floris Taken's well-known and widely implemented 'method of delays' [3]. In this method, a single time series is plotted against a time delayed version of itself in a selected number of dimensions. The time delay is some multiple of the sampling interval. (For experimental time series a minimum sampling interval will exist due to technical constraints of the instrumentation used to make measurements). As a result of the interdependence of the system variables, the fractal dimension of the reconstructed attractor is the same as that of the true attractor, even though there is normally some difference in the shape.

In some experimental systems the number of system variables is unknown, so a series of dimensions must be trialled if the reconstructed attractor is to be clearly plotted. This is the basis for the Grassberger-Proccacia algorithm, where the number of dimensions trialled is referred to as the 'embedding dimension' [4]. All points in the attractor for a time series y_i, with N_s samples, are given by [5]:

$$P_i = (y_i, y_{i+\tau}, y_{i+2\tau}, \ldots, y_{i+(m-1)\tau}); \; i = 1,\ldots,N; \; N = N_s - (m-1)\tau \qquad (1)$$

where m is the embedding dimension and τ is the time delay. There are a number of methods for finding an appropriate value of the time delay, τ. If τ is too small, then the original and delayed time series will be correlated and the points within the attractor will lie along a diagonal in the phase space. This can cause problems when applying various nonlinear analysis techniques. If the delay is too long then the points plotted will be entirely independent of each other and the structure of the attractor will be lost. In this work, τ, has been found from the first

minimum of the mutual information function of each time series [1, 6]. The mutual information function is a measure of how much is known about the delayed vector if we have information about the original time series.

The Grassberger-Procaccia algorithm is used to measure the correlation dimension for a time series [4, 5, 7, 8]. This is just one of many fractal dimension calculation techniques, but is well suited to analysing experimental time series [1]. A phase space attractor is formed for a time series of *N* points using an embedding dimension, *m*. The correlation sum is calculated for several hypersphere radii, *r*, according to the equation:

$$C_m(r) = \frac{2}{N(N-1)} \sum_{i=1}^{N} \sum_{j=i+1}^{N} \theta(r - |P_i - P_j|) \qquad (2)$$

where θ is the Heaviside function. This is equivalent to the density of points within a certain radius, *r*, of each hypersphere. The distances between all pairs of points are measured and only those points with a separation less than *r* are included in the sum. The density of points should scale in a power law with the radius, according to:

$$\lim_{N\to\infty} \lim_{r\to 0} C_m(r) = r^D \qquad (3)$$

Where *D* is the correlation dimension, if such a single value exists. Taking the logarithm of each side gives:

$$D = \lim_{N\to\infty} \lim_{r\to 0} \frac{\log C_m(r)}{\log r}, \qquad (4)$$

which implies when log($C_m(r)$) is plotted against log(*r*), the plot should have a constant gradient, *D*, as $r \to 0$. However, in the analysis of experimental time series, the lack of points and the influence of noise mean that the gradients become unstable at small radii. Also, as *r* increases to the largest point separation in the attractor, the value of log($C_m(r)$) does not rise any further and *D* tends towards zero. Thus, in systems where an attractor with a finite correlation dimension does exist, it is found only from the gradient for an intermediate range of radii.

In this article we have presented plots of *D(r)* (the gradient of log*C*(*r*) versus log(*r*) and referred to hereafter and in the paper as the "gradient") as a function of log(*r*). It is convenient and informative to plot *D(r)*, the gradient, as a function of *r* on a logarithmic scale because the correlation dimension can be found by searching for any horizontal segment of the gradient graph occurring at intermediate radii values [1, 5, 7-9] which should range over several orders of magnitude. The gradient for multiple values of *m* should be plotted on one graph. It is generally accepted that an embedding dimension of up to $m = (2CD + 1)$ should be sufficient for multiple *D(r)* gradient curves, for different *m*, to overlap in a scaling region. But the *CD* is initially unknown [3, 10, 11]. If such a 'plateau' or scaling region exists over a sufficient range of radii, the data can be said to be deterministic with a CD equal to the value of the gradient in that section of the plot.

Since the correlation dimension is a fractal dimension, it provides information about the type of dynamics present in the time series. A value of 1 is found in purely periodic or limit-cycle dynamics, while a value of 2 is found for quasi-periodic data that generates a torus in the phase space. A non-integer correlation dimension indicates the presence of chaotic dynamics in the time series. Systems dominated by noise have no inherent structure and a completely filled

phase space which means that the correlation dimension is effectively infinite. The gradient curves never saturate to a scaling region for heavily noise affected time series, so procedures for reducing noise are recommended where possible.

S1.B A tutorial on finding scaling regions in gradient, *D(r)*, versus radius plots using the minimum gradient detection algorithm

Our procedure for detecting scaling regions in gradient versus radius plots was implemented using MATLAB, following the flow chart shown in Fig A (S1 File). The first step was to load the first time series data file. We analysed the first 10 000 points of each time series from the PIC and SLwOF laser systems, while the time series for the OISSL system were 5000 points long. This substantially reduced the correlation sum calculation time with little effect on the resulting gradient versus radius plots. The mutual information function of the shortened time series was calculated using the *TISEAN 3.0.1 Nonlinear Time Series Analysis* mutual information software [6]. The delay time for the correlation sum calculation was set according to the equivalent integer number of sampling intervals at the first minimum, referred to as the index of the first minimum in mutual information in Fig A (S1 File). To avoid one common source of error in calculating the correlation dimension it is necessary to exclude temporally correlated points from the pair counting (successive points in a time series are usually dependent). A 'Theiler window' was introduced in the calculation, so that points within the window were not included in the correlation sum [12]. In this work, the Theiler window for each time series was chosen to be equal to the number of sampling intervals at the first zero crossing of the autocorrelation function. This was calculated using the inbuilt MATLAB autocorrelation function.

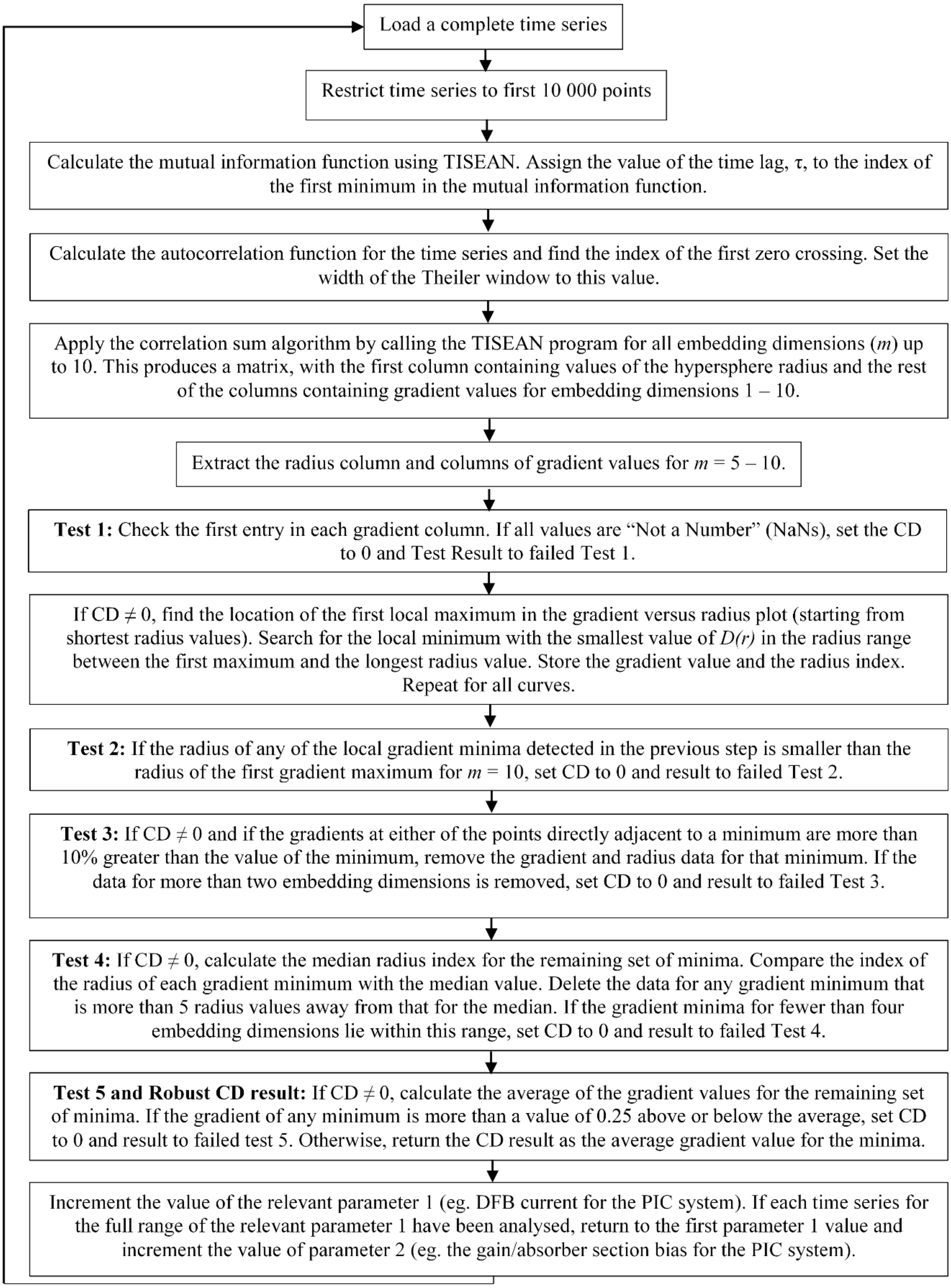

S1 File Fig A. Flowchart of the 'minimum gradient detection algorithm'. The algorithm is for measuring the correlation dimension of time series for a nonlinear system with two system parameters being varied. There are three tolerances inherent to the application of the flow chart of calculations: 1. The percentage difference of gradient values either side of the minimum value at test 3 (10% in the flow chart); 2. The maximum number of radius values away from the median radius of all gradient minima, which each embedding dimension gradient minimum

value can be. This sets the limit for the prospect of a detecting a reasonable scaling region (± 5 radius values in the flow chart); and 3. The range of gradient values for different embedding dimensions taken to be consistent with a scaling region (± 0.25 in the above). Experimentation with the use of smaller or larger values for these tolerances may give useful insights in a CD study of a given system.

The local slopes of the logarithm of the correlation sum were next obtained by running the TISEAN 3.0.1 software package. The maximum embedding dimension was set to 10, with an interval of 1 embedding dimension between successive calculations. The length interval for the hypersphere radii was set to 100 bins, which means that the correlation sum was calculated at 100 different values for the point-to-point separation. The local slopes in the matrix output by the TISEAN software were then smoothed in MATLAB using a 3 point moving average. Since the gradient calculation becomes quite unstable at small radii, all gradients at radii smaller than where the first zero or infinite value was found were set to 'not a number' (NaN) and were not considered in further calculations. Additionally, for this work we only inspected the gradient curves for embedding dimensions between 5 and 10. This was deemed to be a suitable number of dimensions to be able to completely unfold the reconstructed attractors for low-dimensional time series.

The first analysis of the gradient curve data is performed at this point. This is named 'Test 1' and is a check on whether the gradient matrix contains usable data. If the gradients at the longest radius are NaNs for each embedding dimension, then the data fails Test 1. Otherwise, with sufficient gradient data now available for six successive embedding dimensions, the next step in the minimum gradient detection algorithm was to find the first maximum at short radii in each curve. The remaining part of the curves in the direction of the longest radius value were then examined and the gradient value and radius index of the lowest local minima in each curve were recorded. Fig B (S1 File) shows some gradient plots where the lowest local minimum in at least one of the plots for m = 5 – 9 was found at a radius where there was no data for the m = 10 gradient plot. A scaling region does not occur in this data and it was said to fail Test 2.

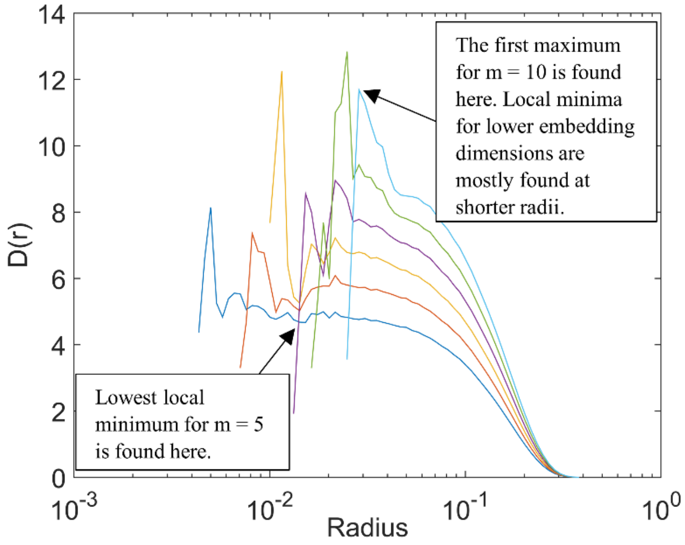

S1 File B. Gradient plots failing Test 2. The potential scaling region at lower embedding dimension has little or no overlap with that at higher dimensions.

Next, the gradients of the points to either side of the local minima are inspected. If the values of these adjacent points are more than a selected threshold value higher than the gradient at the minimum, then the minimum should not be considered to lie in a horizontal scaling region. A value for the correlation dimension is not recorded in this case and the data is listed as failing Test 3. This threshold value can be set to a number of different values when applying the minimum gradient algorithm to interrogate a data set, so that the effect of this on separate mappings can be inspected. Fig C (S1 File) is an example of the gradient plots for a noise affected time series, where the curves are unstable and 'spiky' across a wide range of radii.

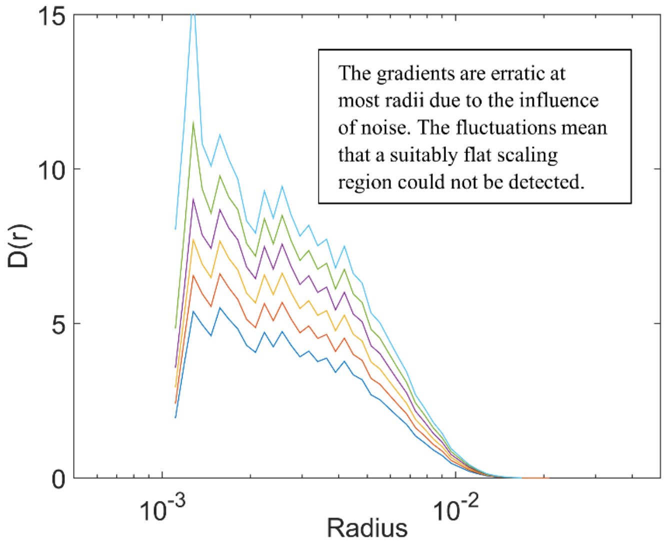

S1 File Fig C. Example of a set of plots failing Test 3 for the lack of fairly flat minima. Also note the increment of 1 between each adjacent plot which is consistent with a noise dominated time series.

The following step in the algorithm determines whether the gradient minima found in each embedding dimension occur for a nearby set of radii. This test was designed to eliminate the effects due to deep spikes at small, noise affected radius values. Alternatively, the curves may have the appearance of Fig D (S1 File), where all minima, for all the embedding dimensions, are detected at intermediate radii, but they are considered to be too far apart. The data fails the algorithm at Test 4. For the test, we determined the median bin number for the radii of the gradient minima. We considered that other minima were at nearby radii only if they were found within five bins either side of the median. The appropriate width is open to variation and should be set by inspection of several gradient plots displaying 'good' scaling regions. Note that it is important to inspect some gradient plots when setting the tolerances for the various tests and as such the computational process should be regarded as semi-automatic rather than automatic.

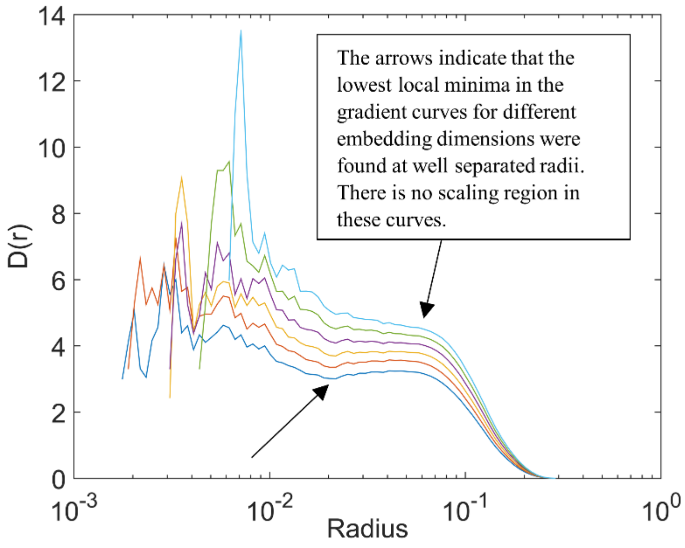

S1 File Fig D. Test 4 is failed. The minima of these curves were considered to be found at too great a radial separation.

The final step in the algorithm checks the vertical spread of the local minimum gradients along the D(r) axis. There is usually some offset in finitely sampled and noisy experimental data, but if this is limited to an increment of approximately 0.1 per embedding dimension, then it is likely that a scaling region is present in the curves [5]. In this paper, the average value of the gradient minima was calculated and a total spread of ±0.25 around the average was allowed. If the minimum from any embedding dimension was outside this range, as in Fig E (S1 File), we considered that a scaling region had not been found. This was recorded as a failure at Test 5. It is to be noted that the increment in the gradient per embedding dimension is significantly less than 1 in the radius range where a near constant gradient is found for all embedding dimensions m = 5-10. Otherwise, the correlation dimension was set to the value of the average and the calculation was considered to be robust. In Fig F (S1 File) the lowest local gradient minima in all embedding dimensions saturate to a tightly bound gradient value and a robust correlation dimension value of 1.27 ± 0.05 is returned by the algorithm.

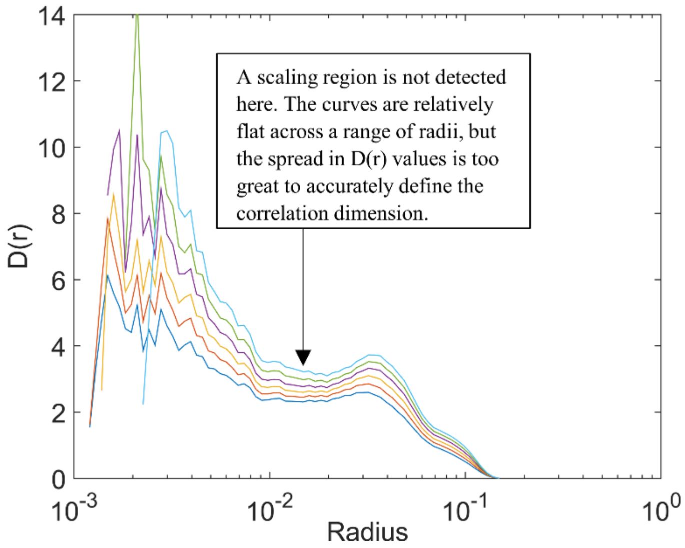

Fig E S1 File. Gradient plots failing Test 5. The vertical spread for the minima in different embedding dimension curves was too great to be interpreted as a scaling region.

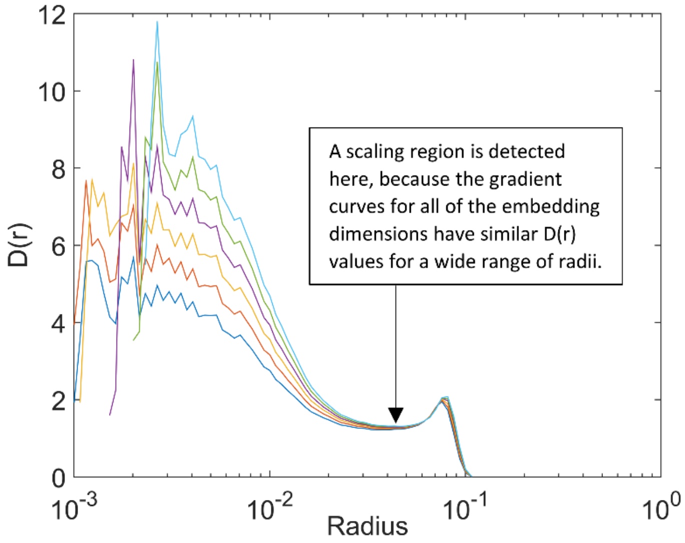

Fig F S1 File. A robust correlation dimension result. The minima are found at similar radii and gradients in the scaling region in these gradient plots.

The test results and the outcome of the correlation dimension measurement were recorded in separate matrices. Test results of 1 – 5 corresponded with test failures. We used a result which passed all tests 1-5 to indicate that a robust correlation dimension was calculated. In the correlation dimension matrix a 0 was used to indicate that a result was not found, although this could also be indicated with another entry, such as a ‘NaN’. The algorithm was performed on each time series by stepping through all parameter 1 (e.g. optical feedback fraction) values for a single parameter 2 value (e.g. injection current), before cycling through the time series for the next highest parameter 2 value.

References

1. Argyris J, Faust G, Haase M, Friedrich R. An Exploration of Dynamical Systems and Chaos. 2nd ed: Springer; 2015.
2. Boon MY, Henry BI, Suttle CM, Dain SJ. The correlation dimension: A useful objective measure of the transient visual evoked potential? Journal of Vision. 2008;8(1). doi: 10.1167/8.1.6.
3. Takens F. Dynamical systems and turbulence. In: Rand DA, Young LS, editors. Springer Lecture Notes in Mathematics: Springer-Verlag, New York; 1981. p. 366-81.
4. Grassberger P, Procaccia I. Characterization of strange attractors. Phys Rev Lett. 1983;50(5):346-9. doi: 10.1103/PhysRevLett.50.346. PubMed PMID: WOS:A1983PZ53000017.
5. Casaleggio A, Bortolan G. Automatic estimation of the correlation dimension for the analysis of electrocardiograms. Biol Cybern. 1999;81(4):279-90. doi: 10.1007/s004220050562.
6. Hegger R, Kantz H, Schreiber T. TISEAN 3.0.1 Nonlinear Time Series Analysis [2/10/2015]. Available from: http://www.mpicks-dresden.mpg.de/~tisean/TISEAN_3.0.1/index.html.
7. Corana A, Bortolan G, Casaleggio A. Most probable dimension value and most flat interval methods for automatic estimation of dimension from time series. Chaos, Solitons & Fractals. 2004;20(4):779-90. doi: http://dx.doi.org/10.1016/j.chaos.2003.08.012.
8. Toomey JP, Kane DM, Valling S, Lindberg AM. Automated correlation dimension analysis of optically injected solid state lasers. Opt Express. 2009;17(9):7592-608. doi: 10.1364/oe.17.007592. PubMed PMID: WOS:000266381700075.
9. Bader R. Nonlinearities and Synchronization in Musical Acoustics and Musical Psychology. 1st ed. Heidelberg: Springer; 2013.
10. Ding M, Grebogi C, Ott E, Sauer T, Yorke JA. Plateau onset for correlation dimension: When does it occur? Phys Rev Lett. 1993;70(25):3872-5.
11. Ding M, Grebogi C, Ott E, Sauer T, Yorke JA. Estimating correlation dimension from a chaotic time series: when does plateau onset occur? Physica D: Nonlinear Phenomena. 1993;69(3–4):404-24. doi: http://dx.doi.org/10.1016/0167-2789(93)90103-8.
12. Theiler J. Efficient algorithm for estimating the correlation dimension from a set of discrete points. Physical Review A. 1987;36(9):4456-62.
